# Testing and treatment for malaria elimination: a systematic review

**DOI:** 10.1186/s12936-023-04670-8

**Published:** 2023-09-03

**Authors:** Gretchen Newby, Chris Cotter, Michelle E. Roh, Kelly Harvard, Adam Bennett, Jimee Hwang, Nakul Chitnis, Sydney Fine, Gillian Stresman, Ingrid Chen, Roly Gosling, Michelle S. Hsiang

**Affiliations:** 1 Malaria Elimination Initiative, Institute for Global Health Sciences, University of California San Francisco (UCSF), 550 16th Street, San Francisco, CA 94143 USA; 2 Department of Women’s and Children’s Health, Uppsala University, Uppsala, Sweden; 3 Department of Epidemiology and Biostatistics, UCSF, San Francisco, CA USA; 4 PATH, Seattle, WA USA; 5 Malaria Branch, Centers for Disease Control and Prevention, U.S. President’s Malaria Initiative, Atlanta, GA USA; 6 Department of Epidemiology and Public Health, Swiss Tropical and Public Health Institute, Allschwil, Switzerland; 7 University of Basel, Basel, Switzerland; 8 College of Public Health, University of South Florida, Tampa, FL USA; 9 Department of Infection Biology, London School of Hygiene & Tropical Medicine, London, UK; 10 Department of Disease Control, London School of Hygiene and Tropical Medicine, London, UK; 11 Department of Pediatrics, UCSF, San Francisco, CA USA

**Keywords:** Malaria, Malaria elimination, *Plasmodium falciparum*, Screen and treat, Test and treat, Active case detection, Proactive, Reactive

## Abstract

**Background:**

Global interest in malaria elimination has prompted research on active test and treat (TaT) strategies.

**Methods:**

A systematic review and meta-analysis were conducted to assess the effectiveness of TaT strategies to reduce malaria transmission.

**Results:**

A total of 72 empirical research and 24 modelling studies were identified, mainly focused on proactive mass TaT (MTaT) and reactive case detection (RACD) in higher and lower transmission settings, respectively. Ten intervention studies compared MTaT to no MTaT and the evidence for impact on malaria incidence was weak. No intervention studies compared RACD to no RACD. Compared to passive case detection (PCD) alone, PCD + RACD using standard diagnostics increased infection detection 52.7% and 11.3% in low and very low transmission settings, respectively. Using molecular methods increased this detection of infections by 1.4- and 1.1-fold, respectively.

**Conclusion:**

Results suggest MTaT is not effective for reducing transmission. By increasing case detection, surveillance data provided by RACD may indirectly reduce transmission by informing coordinated responses of intervention targeting.

**Supplementary Information:**

The online version contains supplementary material available at 10.1186/s12936-023-04670-8.

## Background

Passive case detection (PCD) is the foundation of malaria surveillance and the primary mechanism to detect and treat malaria [1]. However, PCD requires that patients seek care and rates of treatment-seeking behaviour for fever in endemic countries are low, due to limited access to health services [2, 3]. Also, as transmission declines, a larger proportion of malaria infections are low density, and many of these cases will not come to the attention of health facilities due to lack of, or minimal symptoms [1, 4, 5]. Because this reservoir of undetected malaria can perpetuate transmission, these infections are an important target for malaria elimination [6, 7].

To detect infections missed by PCD, active case detection has long been considered core to malaria elimination programs. Broadly, active case detection is applied at mass or targeted geographic scale and may also target demographic groups at high risk of malaria. It may be proactive, directed to areas with known transmission, or reactive, triggered by a recent case usually detected through PCD and directed to areas—typically a defined radius around the household of an index case—or groups with shared risk factors [8]. Active case detection has also been referred to as screen and treat or test and treat (TaT), the latter term used by the World Health Organization (WHO) [9].

Given limited evidence on its effectiveness, in 2015, the WHO recommended against the use of mass or focal TaT using standard diagnostic tests (microscopy and rapid diagnostic tests [RDT]) to reduce transmission [10]. Yet, the 2017 guidelines noted TaT to be an important surveillance component of an elimination strategy [1]. In 2018, the Malaria Elimination Initiative at the University of California, San Francisco conducted an unpublished systematic review of TaT for elimination which included 46 empirical research and 20 modelling studies [11]. Due to continued uncertainty around the role of TaT for malaria elimination and growing literature, the review and results here were updated with the aim of assessing the utility and effectiveness of TaT approaches for malaria transmission reduction and generation of surveillance data to inform elimination strategies.

## Methods

A search on PubMed and Google Scholar was conducted of literature published between January 1900 and October 2021 using selected search terms based on various terminology used for active case detection [12] and a set of exclusion criteria (Additional file 1: Appendix A). Full manuscript reviews were conducted during which additional studies were excluded (n = 148). Included studies were categorized as empirical research or modeling studies. Data regarding study design, year of publication, transmission setting, TaT approaches, and results were collected. If papers included more than one transmission setting, location, or TaT approach, each was treated as a separate study.

### Study type classification

Empirical research studies were categorized by design (intervention or observational), year of publication, eco-epidemiological setting (including location, transmission intensity, *Plasmodium* species), TaT approach, target population (mass versus focal), proactive versus reactive, and diagnostic testing method used. Randomized controlled trials or quasi-experimental studies with comparable controls were classified as intervention studies; pre/post assessments were considered observational studies. If not reported, data on transmission intensity or *Plasmodium* species were collected from contemporaneous studies from the study site.

### Definitions

If the operational unit was a village or larger, studies were classified as mass TaT (MTaT). Studies that targeted sub-village populations were classified as focal TaT (FTaT). Some MTaT or FTaT approaches included socio-demographic high-risk groups. If socio-demographic groups were exclusively targeted proactively, this was referred to as TTaT per WHO nomenclature [9]. Some MTaT interventions were combined with a mass drug administration (MDA)-type intervention. Broadly, MDA refers to drug administration not based on individual level testing. In these studies, MTaT was followed by drug administration to an entire household if any household member tested positive during MTaT, referred to as MTaT + focal MDA (fMDA). Reactive FTaT studies were referred to as reactive case detection (RACD). Transmission intensity categories were based on WHO guidelines (Additional file 1: Appendix B) [1].

### Analysis of intervention studies

For all intervention studies that compared TaT to a control of no TaT, the study design and study-specific effect estimates for incidence and/or prevalence were summarized. Randomized controlled trials were included in an aggregated data meta-analyses to generate a pooled estimate using a random-effects model based on an inverse-variance method. Meta-analyses were conducted using the meta R package [13] and between-study heterogeneity was reported using the *I*^2^ statistic. Some TaT intervention studies compared TaT to a separate MDA-type intervention. Specifically, MTaT was compared to MDA without any testing. Also, RACD was compared to RDA (reactive drug administration), or MDA to an entire household if there was a recent index case (Table 1).

**Table 1:** Test and Treat (TaT), Mass Drug Adminstration (MDA), and their combination

		Mass	Focal	Socio demographic groups only
Proactive	TaT	MTaT^a^	FTaT^b^	TTaT
	MDA	MDA	fMDA	–
	TaT + MDA	MTaT + fMDA	–	–
Reactive	TaT	Reactive MTaT	RACD^c^	–
	MDA	–	RDA	–
	TaT + MDA	–	RACD + RDA	–

MTaT: Mass test and treat; FTaT: Focal test and treat; fMDA: focal mass drug administration; RACD: reactive case detection; RDA: reactive drug administration

In this review, MTaT and FTaT may have also high-risk groups. There were 3 such MTaT studies (see Additional file 1: Appendix E) and 3 such RACD studies (Additional file 1: Appendix F)

^a^Also called mass screen and treat, MSAT

^b^Also called focal screen and treat, FSAT

^c^Also called reactive case detection and treatment, or RACDT

### Analysis of observational studies

For RACD observational analyses, the following surveillance metrics were summarized: (1) yield to detect infection, defined as test positivity rate among individuals screened (using RDT or microscopy versus molecular detection by PCR or loop-mediated isothermal amplification (LAMP)), and (2) relative increase in cases detected using RACD plus PCD versus PCD alone. Studies were pooled by transmission intensity strata and aggregated data meta-analyses were conducted to calculate summary estimates. Reported numbers of RACD and PCD cases detected by LAMP/PCR and/or RDT/microscopy were used to derive study-specific estimates and summary estimates were calculated based on meta-analyses methods described above. RACD arms of intervention studies were included in these analyses.

### Summary of modeling studies

Key findings from TaT modelling studies as they relate to impact on transmission and surveillance were summarized.

## Results

**Fig. 1:**
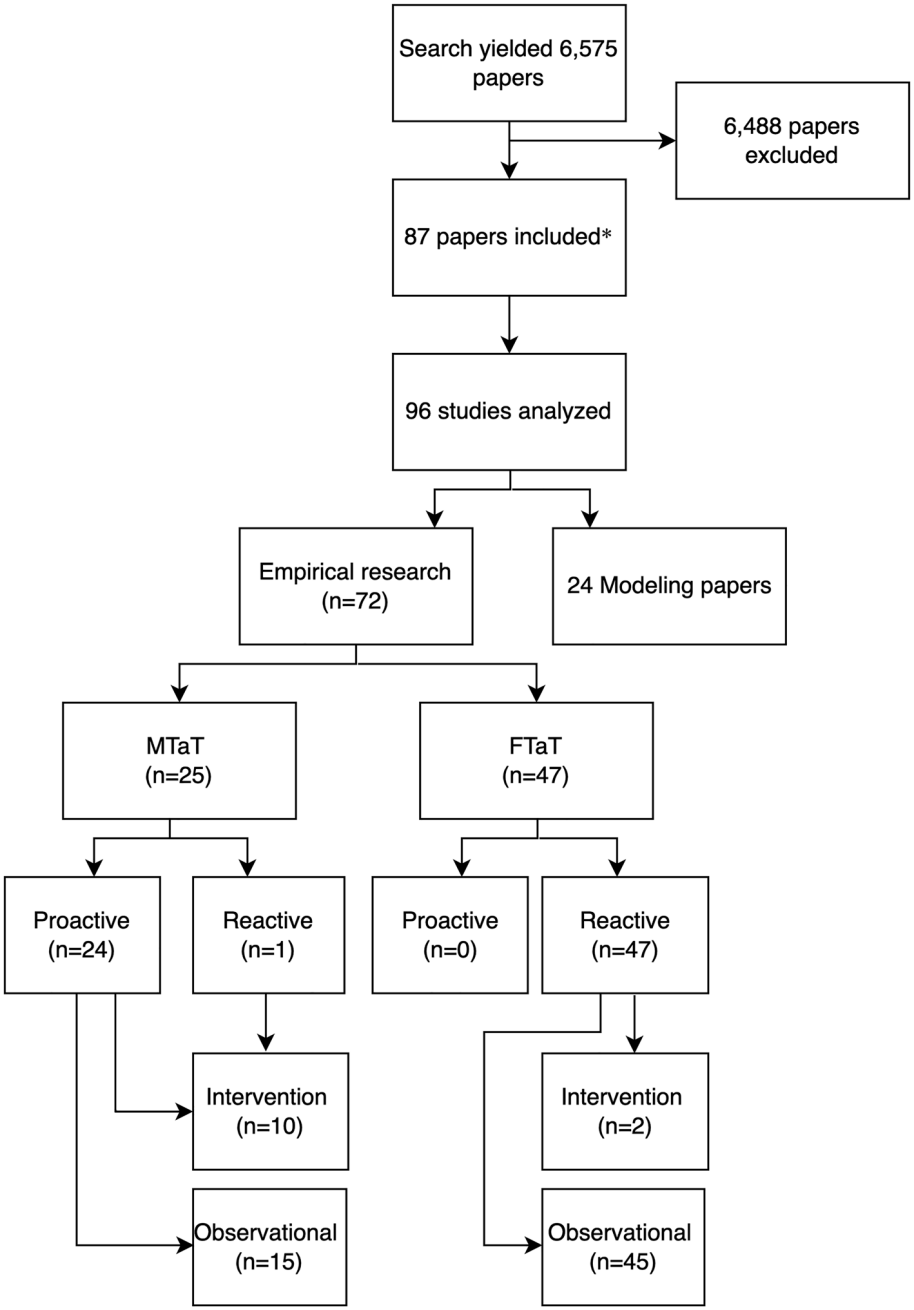
Literature search results. *Eight of the 87 papers included had results from more than one transmission setting, location, or TaT approach and these results were analysed individually; thus, the total number of studies analysed was 96

**Fig. 2:**
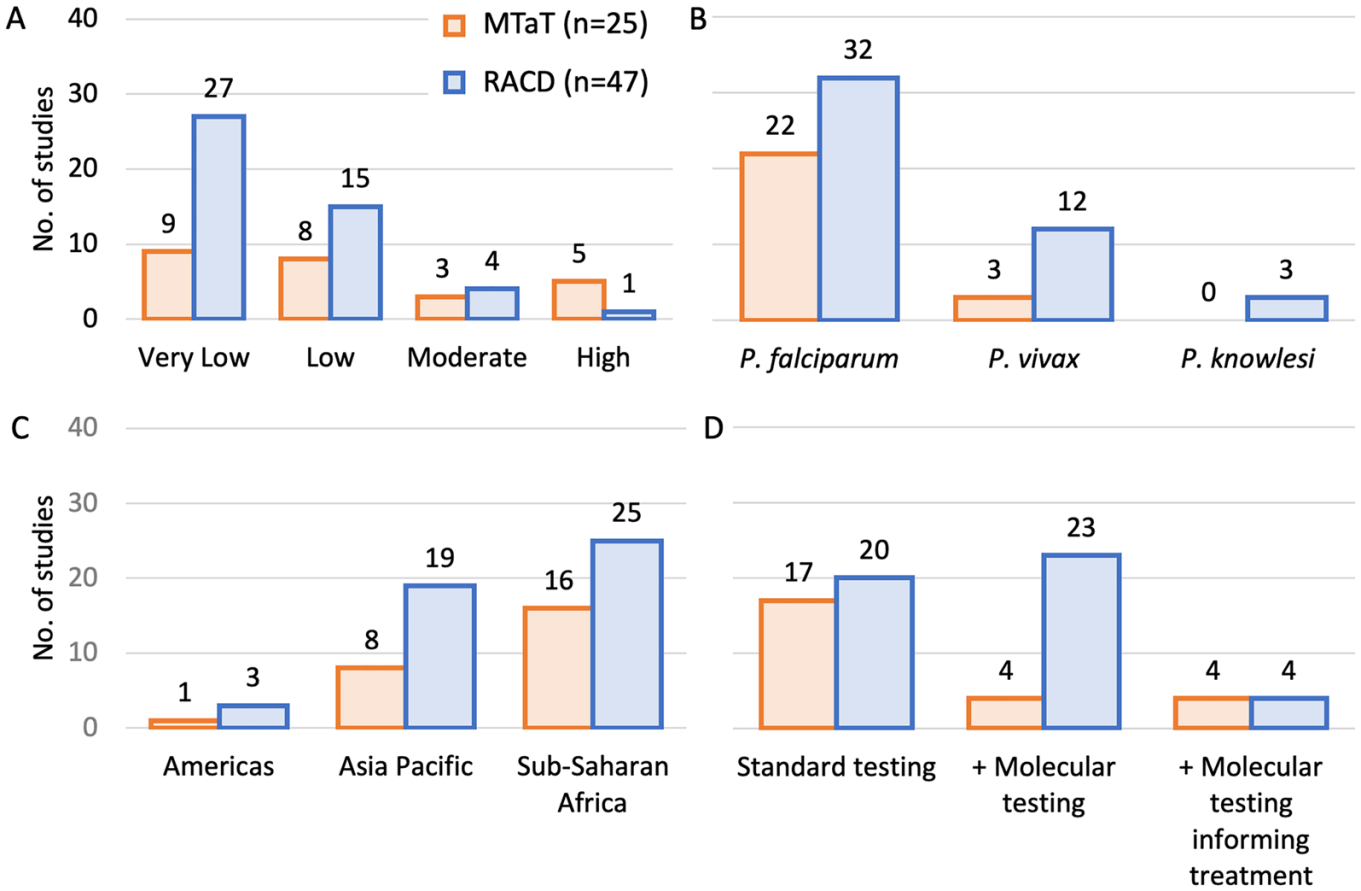
Empiric studies by: **A** Transmission setting, **B** Predominant or targeted *Plasmodium* species, **C** Region, and **D** Diagnostic test used. Standard testing refers to microscopy and/or rapid diagnostic testing (RDT). + Molecular testing refers to standard testing with the addition of molecular testing for surveillance but not to inform treatment. + Molecular testing informing treatment refers to standard testing with the addition of molecular testing for both surveillance and to inform treatment

The literature search yielded 6,575 papers, and based on review of their titles and abstracts, 87 were selected for inclusion (Additional file 1: Appendix C). Eight of the 87 presented results from more than one transmission setting, location, or TaT approach, and these were subdivided into 96 separate studies. Of these, there were 72 empirical research studies and 24 modelling studies (Fig. 1). Empirical research studies were of MTaT (n = 25) or FTaT (n = 47). Almost all MTaT studies were proactive, except for one reactive study [14]. All FTaT studies were reactive, referred to as RACD, and targeted geographic areas at the sub-village level and/or individuals based on socio-demographic risk factors. Some MTaT studies also included TTaT but here were no empirical research studies of TTaT alone. Of the 12 intervention studies, 10 focused on MTaT and 2 on RACD; all other studies were observational (Fig. 1). The first study was published in 2005 and the annual number of papers increased since 2014 (Additional file 1: Appendix D). Most studies were from sites with lower transmission in sub-Saharan Africa or the Asia Pacific region, with *Plasmodium falciparum* as the predominant species. Eight studies used molecular testing to inform treatment (Fig. 2).

### MTaT intervention studies

The MTaT intervention studies were proactive MTaT (n = 6) [15–20], proactive MTaT with focal MDA (MTaT + fMDA) with treatment administered to an entire household or compound if there was at least one positive individual within a sentinel population (n = 3) [21, 22], and reactive MTaT (n = 1) (Table 2) [14]. The trials were largely from *P. falciparum*-predominant settings in Africa, and all were cluster designs with total number of clusters ranging from 4 to 30. MTaT was implemented up to 3 times annually and coverage levels varied. Only two of the ten trials showed an impact on prevalence. In a randomized trial of MTaT + fMDA from Zambia, prevalence was only assessed in children < 5 years and a smaller reduction was seen on incidence [16]. The other, a quasi-experimental study from Tanzania evaluated a rolling reactive MTaT approach whereby each week the village with the highest incidence in the previous week was targeted, but the study was limited by risk of selection bias, a small sample size (n = 4 clusters), and coverage was not reported [14]. Other MTaT or MTaT + fMDA studies attributed non-statistically significant impact with high transmission intensity [18], missed infection due to poor coverage and/or human movement [15, 19–21], and limited sensitivity of the diagnostic [15, 17, 18, 21, 22].

**Table 2:** Mass test and treatment (MTaT) versus no MTaT intervention studies

Study	Country	Transmission	Species	TaT method	Proactive or Reactive	Design	Total no. clusters^c^	Total population	Test	Drug regimen	No. of rounds or events	MTaT coverage	Outcome for MTaT versus no MTaT				
													Prevalence		Incidence		
													Follow up (months)	Infection, OR, 95% CI^a^	Follow up (months)	Symptomatic cases, IRR, 95% CI^b^	Infection, IRR, 95% CI^b^
Desai et al*.* 2020	Kenya	High	Pf	MTaT	Proactive	CRCT	30^c^	21–50K	RDT/M	DP	6	75–94%	–	–	24	0.79, 0.61–1.02	0.95, 0.87–1.04
Eisele et al*.* 2016	Zambia	High	Pf	MTaT + fMDA	Proactive	CRCT	20	> 50K	RDT	DP	2	54–63%	12	0.57, 0.13–2.50	5	0.97, 0.73–1.29	0.75, 0.31–1.78
Samuels et al*.* 2021	Kenya	High	Pf	MTaT	Proactive	CRCT	20	21–50K	RDT	DP	6	75–94%	21	0.92, 0.76–1.10Δ	–	–	–
Tiono et al*.* 2013	Burkina Faso	High	Pf	MTaT	Proactive	CRCT	18	5–20K	RDT	AL	3	96%	12	0.92, p = 0.3^a^	12	1.06, p = 0.3	–
Larsen et al*.* 2015	Zambia	Moderate	Pf	MTaT	Proactive	CRCT	16	> 50K	RDT	AL	3	88%	12	0.47, 0.24–0.90^a^	12	0.83, 0.68–1.01	–
Mlacha et al*.* 2020	Tanzania	Low/Moderate	Pf	MTaT	Reactive	CQED	4	> 50K	RDT	DP	85^d^	Not reported	28	0.34, 0.26–0.44	13	–	–
Bousema et al*.* 2016	Kenya	Low	Pf	MTaT + fMDA	Proactive	CRCT	10	< 5K	RDT	AL	2	94%	4	difference 1.0, − 8.3–10.4^a^	–	–	–
Eisele et al*.* 2016	Zambia	Low	Pf	MTaT + fMDA	Proactive	CRCT	20	> 50K	RDT	DP	2	54–63%	12	1.28, 0.36–4.60	5	0.80, 0.60–1.08	0.77, 0.22–2.71
Sutanto et al*.* 2018	Indonesia	Low	Pv > Pf	MTaT	Proactive	CRCT	16^c^	< 5K	M	DP + PQ	3	89%	–	–	5	–	1.04, 0.36–2.98^b^
											2	87%					0.99, 0.62–1.59^b^
Cook et al*.* 2015	Zanzibar	Very Low	Pf	MTaT	Proactive	CQED	10	21–50K	RDT	ASAQ	2	43–54%	–	–	6	No difference^e^	–

Studies listed in bands from highest to lowest transmission intensity setting, and alphabetically by author within each band

Desai et al. and Samuels et al. were part of the same trial, but presented separately due to different sample sizes used in the two papers

Pf *Plasmodium falciparum*; Pv *Plasmodium vivax*; MTaT mass test and treat; fMDA focal mass drug administration; CQED cluster quasi-experimental design; CRCT cluster randomized controlled trial; K thousand; RDT rapid diagnostic test; M microscopy; AL artemether–lumefantrine; DP dihydroartemisinin–piperaquine; PQ primaquine; ASAQ artesunate-amodiaquine; OR odds ratio; CI confidence interval; IRR incidence rate ratio

^a^Assessed in all ages and by RDT, except for Larsen et al. (in children < 5 years of age and using RDT), Tiono et al. (in children < 5 years of age with parasite density > 5000 p/μL by PCR), and Bousema et al*.* (all ages, by PCR)

^b^Assessed in children and by RDT or microscopy, except for Sutanto et al. where also assessed by PCR (results for both studies not statistically significant and not shown) and Tiono et al. where assessed in all ages

^c^Numbers of intervention and control clusters were equal, except Sutanto et al. (3 rounds of MTaT in 6 clusters, 2 rounds of MTaT in 5 clusters, 5 control clusters) and Desai et al. (10 intervention clusters, 20 control clusters)

^d^Weekly rounds of MTaT (n = 85 over study period) targeted to villages with the highest recent test positivity rates

^e^Estimates not provided

^f^Adjusted ratio of prevalence ratios

**Fig. 3:**
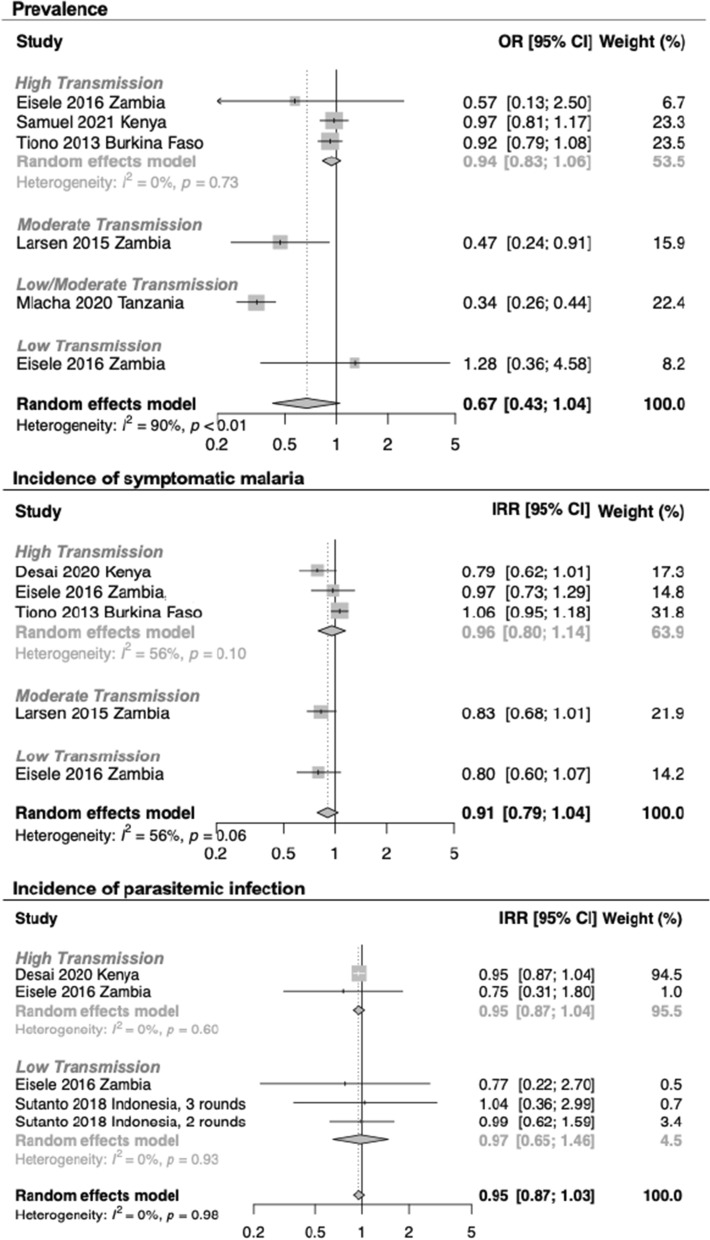
Meta-analysis of intervention studies evaluating mass test and treat (MTaT) versus no MTaT

Among the randomized controlled trials included in the MTaT meta-analysis, there may have been information bias, especially because the nature of the MTaT intervention made blinding impossible. For the incidence assessment, perceptions that MTaT was effective may have influenced participant care-seeking and provider management of fever, leading to under-reporting. It is also possible that there was a bias toward the null if MTaT led to increased detection of incident cases, due to increased vigilance among the population and/or providers. For the prevalence assessment, reporting bias was unlikely as testing was not dependent on care-seeking. However, observer bias was possible as most studies did not provide data to show that the population sampled in cross-sectional surveys was representative of the total population (e.g. % not reached by arm). Summary estimates from meta-analyses (Fig. 3) found that MTaT was associated with minimal impacts on prevalence (OR=0.67 [95% CI 0.43, 1.04]) symptomatic malaria (IRR = 0.91 [95% CI 0.79, 1.04]) and incidence of parasitaemia (IRR = 0.95 [95% CI 0.87, 1.03]). Two MTaT + fMDA studies from Zambia additionally evaluated community-wide MDA versus control and found it reduced prevalence and incidence in a low transmission area [21].

### MTaT observational studies

There were 15 proactive MTaT observational studies that examined impact on transmission, yield, and operational feasibility, including implementation of different diagnostic approaches and TTaT as part of MTaT. The studies that aimed to assess impact were pre-post assessments limited by bias and confounding [23–28]. Significant operational challenges of MTaT, whether implemented by standard (microscopy or RDT) or molecular testing were noted (Additional file 1: Appendix F).

### FTaT intervention studies

There were no proactive FTaT intervention studies. There were two RACD intervention studies from *P. falciparum*-predominant settings in southern Africa (Table 3) [29, 30]. As it was already a standard of care intervention, RACD was not compared to a control of no RACD. Rather, RACD was the control, and was compared to reactive drug administration (RDA) alone (also referred to in the literature as reactive focal MDA, or rfMDA) or in combination with reactive indoor residual spraying. In a low transmission setting in Namibia, RDA and RDA + reactive indoor residual spraying decreased prevalence and incidence [29]. In another study from a very low transmission setting in Eswatini, RACD was compared to RDA [30]. The strength of the evidence to suggest impact on incidence was weak, and this was attributed to low coverage and limited statistical power.

**Table 3:** Reactive case detection (RACD) versus reactive drug administration (RDA) intervention studies (n = 2)

Study	Country	Trans-mission	Species	Design	Total no. clusters	Total population	Test	Drug regimen							Outcome					
									RACD as control		RDA Intervention		RDA + RAVC Intervention		Prevalence of infection			Incidence of symptomatic cases		
									No. of events	Coverage of events	No. of events	Coverage of events	No. of events	Coverage of events	Follow up (months)	RDA vs RACD PR, 95% CI	RDA + RAVC vs RACD PR, 95% CI	Follow up (months)	RDA vs RACD IRR, 95% CI	RDA + RAVC vs RACD IRR, 95% CI
Ntuku et al*.* 2020	Namibia	Low	Pf	CRCT	56	5–20K	RDT	AL	178	84%	164	91%	144	89%	8	0·59 (0·21–0·98)	0·16 (0·05–0·55)	12	0·52 (0·16–0·88)	0·26 (0·10–0·68)
Vilakati et al*.* 2021	Eswatini	Very low	Pf	CRCT	77	> 50K	RDT	DP	46	70%	64	62%	–	–	–	–	–	24	0.84 (0.42 to 1.66)	–

RACD reactive case detection, RDA reactive drug administration; RAVC reactive vector control (indoor residual spraying using pirimiphosmethyl)

Prevalence assessed in all ages and using PCR

Incidence of symptomatic local cases assessed in all ages through passive case detection

### FTaT observational analyses

There were 45 FTAT observational studies, all on RACD. The RACD arms of the two FTaT intervention studies [29, 30] were additionally included in this analysis. Most were from low and very low transmission settings and non-*falciparum* species were predominant in approximately half (Additional file 1: Appendix F and Fig. 2). RACD was generally triggered in response to recent symptomatic, locally acquired, laboratory-confirmed cases passively detected at health facilities or within communities and targeted members of the index case household and neighbouring households. The extent of screening beyond the index household was reported as maximum radius (range 100–3000 m) or number of households (range 4 to 10), and based on local factors, including maximum flight range for *Anopheles* mosquitoes [31, 32], local data regarding clustering of infections [33–35], population density [33, 36, 37], ecological conditions facilitating local transmission [32, 38–40], and logistical constraints [32, 36, 41–43]. Possibly due to the latter, four studies included initial screening for subjects with fever [43–46], though most RACD-identified infections were afebrile. Goal response times were 2–28 days (median 7 days). With the exception of one study that conducted RACD including primaquine use in four rounds over 180 days to maximize detection of infections, including *Plasmodium vivax* relapses [31], all studies reported one round of RACD. Several studies reported operational challenges with coverage and response time [32, 41, 47–49] but most studies did not monitor or report these figures. Few studies (n = 4) utilized molecular testing to inform treatment. Methods with high throughput (e.g., PCR pooling), or rapid amplification (e.g., LAMP) were used, and turnaround time for results was 13 days [50–52] or < 7 days [9] [personal communication].

There was a positive relationship between higher transmission intensity and RDT- or microscopy-positivity rates among individuals tested in RACD: overall RACD positivity was 85.9% [95% CI 81.3, 89.7], 9.6% [95% CI 3.3, 24.5], 4.4% [95% CI 2.2, 8.3], and 0.6% [95% CI 0.4, 0.8] in high, moderate, low, and very low transmission settings, respectively (Additional file 1: Appendix G). More sensitive molecular testing was included in 27 studies, all of which were from low or very low transmission settings. Molecular methods increased detection of infections by 2.2-fold [95% CI 1.8, 2.6] and 2.8-fold [95% CI 2.5, 3.2] in low and very low transmission settings, respectively (Additional file 1: Appendix H).

**Fig. 4:**
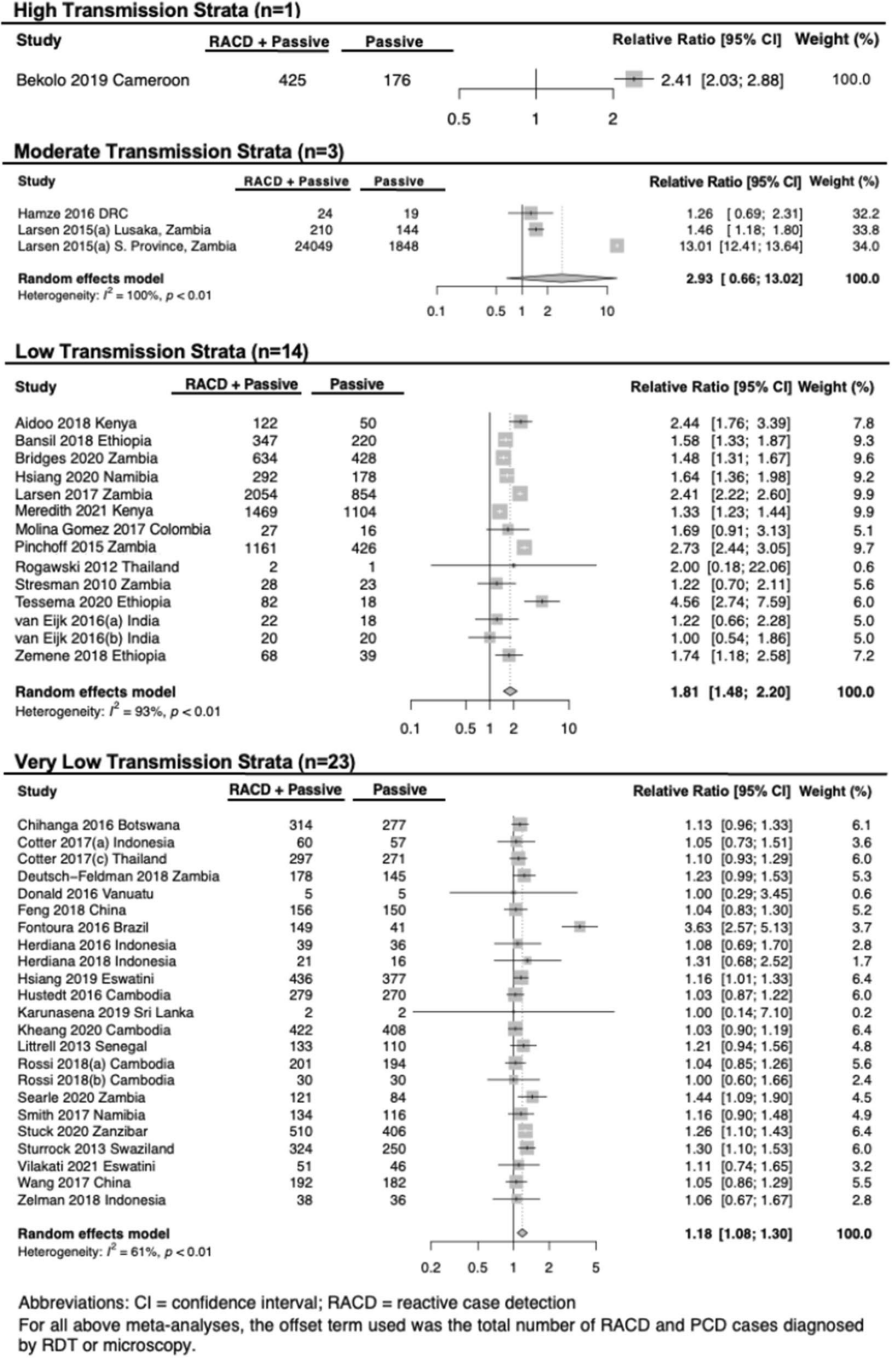
Relative difference in number of cases by RACD using RDT/ microscopy + passive case detection (PCD) versus PCD alone. Summary estimates are reported by transmission strata and generated using a random effects model. Relative Ratio only takes into consideration PCD cases which lead to RACD, which, in most studies, were all PCD activities. This measure may be lower than if PCD activities that did not lead to RACD were also included, such as in Larsen et al. where PCD leading to RACD was reported as 1848, whereas the total number of PCD identified cases was 53,463

**Fig. 5:**
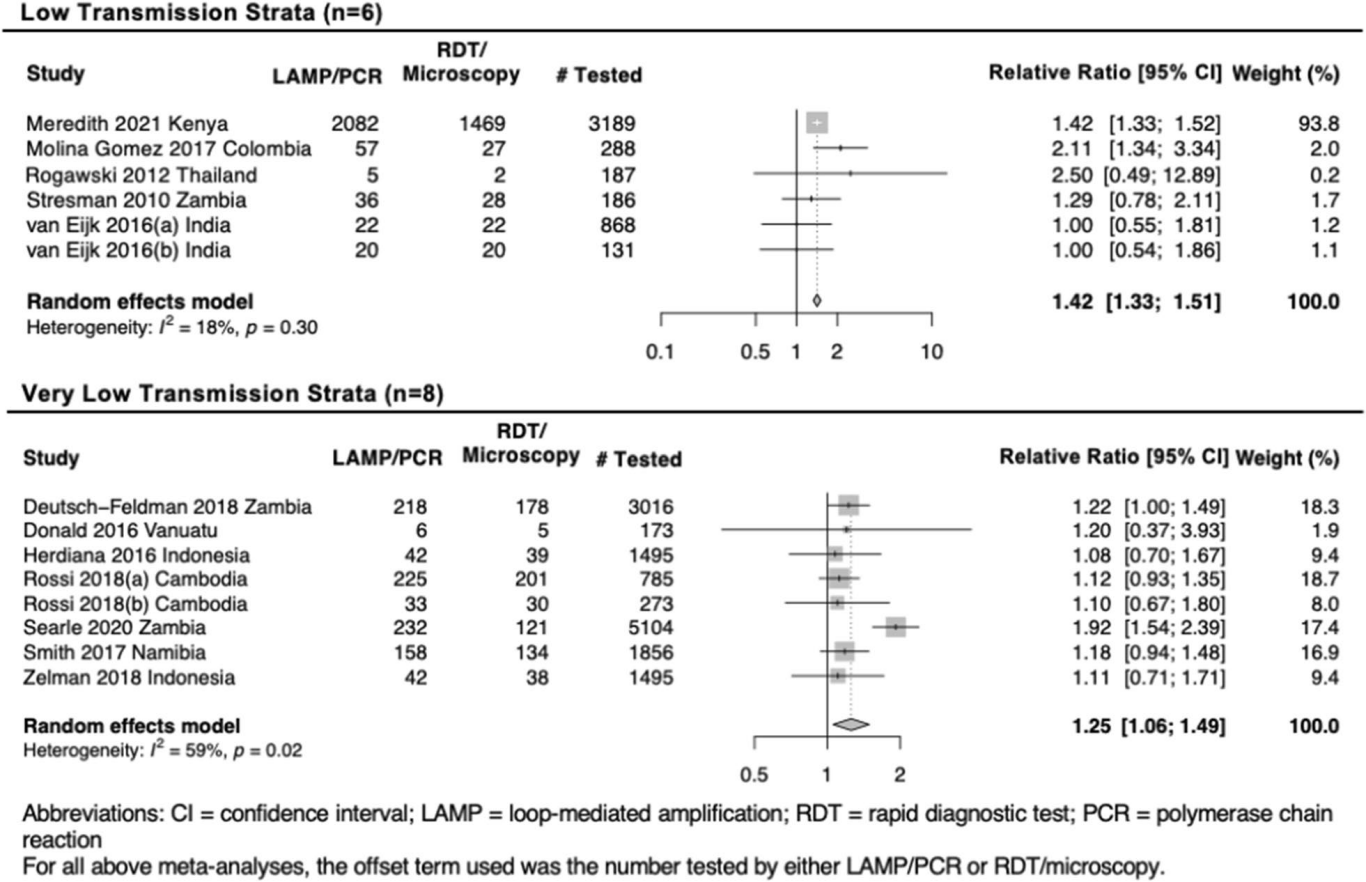
Relative difference in number of cases found by RACD using LAMP/PCR + PCD versus RACD using RDT/microscopy + PCD. Only includes studies where all reactive case detection (RACD) individuals were tested by both LAMP/PCR and RDT/microscopy. Summary estimates are reported by transmission strata and generated using a random effects model

In 44 studies, the number of passively detected index cases triggering RACD was reported, enabling us to report a relative increase in the number of cases detected by RACD + PCD versus PCD alone. As with test positivity rate among individuals screened, this value correlated with transmission intensity. RACD using RDT/microscopy + PCD versus PCD alone was associated with an increase in detection of RDT- or microscopy-cases by 2.4-fold [95% CI 2.0, 2.9], 2.9-fold [95% CI 0.7, 13.0], 1.8-fold [95% CI 1.5, 2.2], and 1.2-fold [95% CI 1.1, 1.3] in high, moderate, low, and very low transmission settings, respectively (Fig. 4). Of the studies that also tested the same RACD individuals using molecular methods, the increase in number of cases detected with RACD using LAMP/PCR + PCD compared to PCD alone was 1.4- and 1.3-fold in low and very low transmission settings, respectively (Fig. 5).

Eight RACD studies reported testing index case contacts based on co-travel [45] or similar high-risk exposures such as occupation (Additional file 1: Appendix F) [50–54]. Only one of these studies, from Cambodia, compared test positivity rates of this approach to standard RACD among index case household members and neighbors and found that the former yielded a higher positivity rate [50]. Three RACD studies also conducted TTaT; one (also from Cambodia) compared TTaT to RACD targeting domestic and non-domestic co-exposed contacts and found that the former was higher yield in terms of number of additional cases detected [49].

### Modelling studies

Twenty-four mathematical and simulation modelling studies assessing the impact and efficiency of TaT strategies were identified. Most (n = 21) were focused on Africa, primarily Zambia (n = 10); only three modelling studies focused on non-African geographies [55–57]. Studies were of MTaT (n = 12), RACD (n = 8), and TTaT (n = 4 on border screening targeting visitors and returning residents).

MTaT modelling studies examined the roles of coverage, timing, frequency, diagnostic sensitivity, and transmission intensity. It was generally found that increasing (1) coverage, (2) number of MTaT rounds during the dry season, (3) years of implementation, and (4) diagnostic sensitivity improved the effectiveness of MTaT to reduce transmission, and impact was more sustained in low transmission settings [57–64]. Implementing MTaT in combination with moderate to high coverage of other interventions such as vector control and case management led to greater transmission reduction [61, 62, 65, 66]. Five studies modelled the impact of MTaT compared to MDA, and all determined that MDA was more effective than MTaT in reducing transmission due to limited infection detection of standard diagnostics and MDA’s prophylactic in addition to treatment effect [60, 63, 64, 67, 68]. In settings with high clustering of infections, MTaT + focal MDA (fMDA) was more effective than MTaT alone, though the optimal diagnostic method used in MTaT varied [59, 63, 67, 68].

Four studies modeled the impact of border screening at entry points in Lao PDR and South Africa [56, 69–71]. *Plasmodium falciparum* transmission was reduced, but elimination could only be achieved when implemented as a component of a comprehensive package of interventions including vector control and MDA.

There were eight RACD modelling studies, of which seven were from Zambia [63, 72–77] and one from Myanmar [55]. Implemented at very high coverage, RACD was predicted to decrease transmission, but not be operationally or financially feasible for many programmes [55, 73–77]. RACD utilizing more sensitive diagnostics to detect infection or past exposure [63, 75] or RDA as an alternative to RACD showed promise [75, 76]. Imported malaria was seen as a barrier to the effectiveness of RACD [55, 75], though a recent study found that RACD could help prevent onward transmission of imported infections [76]. Relapses were an additional identified challenge in the only RACD modeling study from a *P. vivax* endemic setting [55].

## Discussion

There is a high level of interest regarding the role of TaT for malaria control and elimination. The studies identified in this systematic review and meta-analysis of TaT strategies for malaria elimination largely focused on proactive MTaT in higher transmission settings and RACD in lower transmission settings. The strongest available evidence suggested that MTaT using RDTs or microscopy had minimal impacts on prevalence and incidence and findings from modeling studies were consistent. The effectiveness of RACD for transmission reduction could not be reviewed due to no intervention studies comparing RACD to a control of no RACD. However, the utility of RACD for surveillance was assessed by measuring yield for infection detection. Across transmission settings, PCD + RACD, especially with molecular diagnostics, increased detection of infections compared to PCD alone. This finding, along with strong evidence that infections cluster around index cases, suggest that RACD has utility for providing surveillance data that can lead to a coordinated response of interventions.

Based on a separate review that relied largely on controlled studies, the WHO recently issued new guidelines on test and treat strategies for malaria elimination [9]. This study, as an independent review of TaT, and with the additional inclusion of observational and modelling studies, and hybrid MTaT/MDA approaches, provides a useful context for reviewing these new guidelines. For proactive MTaT, findings from the WHO review were similar and MTaT was not recommended [78]. This study additionally included other MTaT approaches (MTaT + fMDA and reactive MTaT) where drug-associated costs and risks are limited to a subset of higher risk individuals, and found that they are also unlikely to decrease transmission.

Similar to this study, the WHO review did not identify any trials that assessed the impact of RACD versus no RACD on transmission reduction, but considered two pre-post assessments that showed no [31] or minimal impact [79, 80]. Nonetheless, the WHO guidelines endorse RACD for transmission reduction and for surveillance in the end-stage of an elimination programme. This study augments the literature by providing an analysis of surveillance metrics from 47 RACD studies. This study identified increased detection of infection for RACD using molecular testing versus standard diagnostics and provides a review of the evidence on PCD + RACD versus PCD alone. While the percent increases were greater in higher transmission settings, the modeling studies suggested that the operational challenge of RACD precludes feasible implementation in most settings, with the exception of very low transmission settings. Regarding molecular testing used in RACD, the long turn-around times and few studies using results to inform treatment suggest operational challenges. But such surveillance information could inform subsequent targeting of interventions (e.g., drug administration, vector control, or vaccines), which could then lead to transmission reduction. Further, these samples could be used for genomic analysis to ascertain the extent to which RACD can interrupt transmission networks [81].

Due to clustering of infections [82] in and around households, RACD typically targets households in that transmission is often peri-domestic. RACD targeting socio-demographic high risk groups is also endorsed in the recent WHO guidelines, but without accompanying evidence. Although several RACD studies targeted such groups, all but one [50] aggregated with data from peri-domestic targeting, precluding comprehensive analysis of this approach. The WHO review found limited evidence regarding proactive targeting of socio-demographic groups in the community (TTaT) or at borders (border screening), and recommended against these approaches. In this study TTaT was used alongside RACD and MTaT, but studies were few, thus precluding an analysis of the approach [50–52]. Additionally, analyses of border screening in two observational MTaT studies [83, 84] and four modelling studies suggested benefit of this approach. Further research on TaT targeting high risk groups are needed.

Other evidence gaps or limitation were identified. First, findings may not be generalizable across regions and transmission intensities. Most of the studies were from sub-Saharan Africa where transmission dynamics, *Plasmodium* species, and characteristics of high-risk groups are quite different from the Americas or Asia–Pacific region. Also, transmission intensity groupings may have masked the role of confounding factors such as vector behaviour and ecology. Second, there was minimal evidence regarding monitoring and evaluation and quality improvement for the various TaT strategies across all geographies, making it difficult to standardize assessments of quality and impact and compare results across studies. Third, except for MTaT, there were few trials. Cluster randomized controlled trials will provide the strongest quality of evidence, but trials of RACD versus no RACD are unlikely to be conducted since RACD is standard practice. Also, many evidence gaps regarding the impact of RACD on transmission reduction are likely to be addressed in more feasible study designs such as quasi-experimental studies (e.g., interrupted time series analyses), ring trials for reactive or focal interventions [85], or analyses of high-quality surveillance data, including genomic analyses.

While this study was not focused on MDA, if TaT was compared to MDA, these findings were reported. MDA was used as a comparison to TaT in modelling studies, and whether delivered to entire communities or in targeted or reactive approaches, it was generally more effective than test and treat methods due to diagnostic challenges and prophylactic effect. In empirical studies, TaT was only directly compared to MDA in the two RACD intervention studies (RACD vs RDA) [29, 30]. Despite low certainty of evidence for RDA versus RACD, WHO’s recommendation in favor of RDA is based on consideration that effect of RDA was likely underestimated due to potential effect of RACD. The WHO’s recommendations for MDA and targeted drug administration (TDA) are based on comparisons of MDA to no MDA, and TDA to no TDA [9], both of which were beyond the scope of this study. However, this study captured some hybrid TaT + MDA approaches, which may address safety and drug resistance risks associated with MDA [86]. Some modelling studies have attempted to identify the optimal scenarios for TaT versus MDA versus blended approaches (e.g. MTaT + fMDA or RACD + fMDA) [63], but a simpler framework that has relevance to a range of geographies is needed [87].

This study emphasizes the growing importance of TaT strategies, provides additional nuance to existing WHO guidance on the potential role of these strategies in accelerating elimination, and identifies several opportunities for further research to guide policy.

### Supplementary Information

**Additional file 1.** Appendix A: Literature search terms and exclusion criteria. Appendix B: WHO categories of malaria transmission intensity. Appendix C: Studies assessed for inclusion in the review (n=235). Appendix D: Cumulative number of studies published by year. Appendix E. Summary of observational proactive MTaT studies (n=15). Appendix F: Details of intervention and observational RACD studies (n=47). Appendix G: Reactive case detection (RACD) Positivity (%) by Rapid diagnostic test (RDT) or microscopy. Appendix H: Relative difference in reactive case detection (RACD) positivity by LAMP/PCR versus RDT/microscopy. Summary estimates are reported by transmission strata and generated using a random effects model.

## Data Availability

The datasets used and/or analysed will be available from the corresponding author upon reasonable request.
