# Correction: Testing and treatment for malaria elimination: a systematic review

**DOI:** 10.1186/s12936-024-04861-x

**Published:** 2024-03-01

**Authors:** Gretchen Newby, Chris Cotter, Michelle E. Roh, Kelly Harvard, Adam Bennett, Jimee Hwang, Nakul Chitnis, Sydney Fine, Gillian Stresman, Ingrid Chen, Roly Gosling, Michelle S. Hsiang

**Affiliations:** 1 Malaria Elimination Initiative, Institute for Global Health Sciences, University of California San Francisco (UCSF), 550 16th Street, San Francisco, CA 94143 USA; 2 Department of Women’s and Children’s Health, Uppsala University, Uppsala, Sweden; 3 Department of Epidemiology and Biostatistics, UCSF, San Francisco, CA USA; 4 PATH, Seattle, WA USA; 5 Malaria Branch, Centers for Disease Control and Prevention, U.S. President’s Malaria Initiative, Atlanta, GA USA; 6 Department of Epidemiology and Public Health, Swiss Tropical and Public Health Institute, Allschwil, Switzerland; 7 University of Basel, Basel, Switzerland; 8 College of Public Health, University of South Florida, Tampa, FL USA; 9 Department of Infection Biology, London School of Hygiene & Tropical Medicine, London, UK; 10 Department of Disease Control, London School of Hygiene and Tropical Medicine, London, UK; 11 Department of Pediatrics, UCSF, San Francisco, CA USA

**Correction: Malaria Journal (2023) 22:254** 10.1186/s12936-023-04670-8

Following publication of the original article [1], the authors flagged two sets of errors (one in the Results and the other in Table 2) and made one clarification (in the caption of Figure 4): The first error arose because they updated Fig. 3 prior to submission but neglected to update the accompanying text; the second error occurred when transferring information from one document to another; the clarification has been made because of concern that the relative ratios presented in Figure 4 may be subject to misinterpretation. To address these concerns, the following changes have been made to the published article:

Error #1

The original text in the Results:

- “Summary estimates from meta-analyses (Fig. 3) found that MTaT was associated with minimal impacts on prevalence (OR = 0.92 [95% CI 0.82, 1.03])…”

The corrected text in the Results:

- “Summary estimates from meta-analyses (Fig. 3) found that MTaT was associated with minimal impacts on prevalence (OR = 0.67 [95% CI 0.43, 1.04])...”

Error #2.

The original text in Table 2:

- “Cook et al. 2015 | Zanzibar | Very Low | … | TaT Rounds |”

The corrected text in Table 2:

- “Cook et al. 2015 | Zanzibar | Very Low | … | 2 |”

Clarification:

Added to the end of Figure 4 caption:

- “Relative Ratio only takes into consideration PCD cases which lead to RACD, which, in most studies, were all PCD activities. This measure may be lower than if PCD activities that did not lead to RACD were also included, such as in Larsen et al. where PCD leading to RACD was reported as 1848, whereas the total number of PCD identified cases was 53,463.”

The authors thank you for reading this erratum and apologize for any inconvenience caused.
