# Supplementary material for: Testing and treatment for malaria elimination: a systematic review
Source: Malar J. 2023 Sep 3;22:254. doi: 10.1186/s12936-023-04670-8 (PMC10476355; doi:10.1186/s12936-023-04670-8)
Supplement: Supplementary file 1 — Additional file 1. Appendix A: Literature search terms and exclusion criteria. Appendix B: WHO categories of malaria transmission intensity. Appendix C: Studies assessed for inclusion in the review (n=235). Appendix D: Cumulative number of studies published by year. Appendix E. Summary of observational proactive MTaT studies (n=15). Appendix F: Details of intervention and observational RACD studies (n=47). Appendix G: Reactive case detection (RACD) Positivity (%) by Rapid diagnostic test (RDT) or microscopy. Appendix H: Relative difference in reactive case detection (RACD) positivity by LAMP/PCR versus RDT/microscopy. Summary estimates are reported by transmission strata and generated using a random effects model. [file 12936_2023_4670_MOESM1_ESM.docx]

**Appendices**

Appendix A: Literature search terms and exclusion criteria

Appendix B: WHO categories of malaria transmission intensity

Appendix C: Studies assessed for inclusion in the review (n=235)

Appendix D: Cumulative number of studies published by year

Appendix E. Summary of observational proactive MTaT studies (n=15)

Appendix F: Details of intervention and observational RACD studies (n=47)

Appendix G: Reactive case detection (RACD) Positivity (%) by Rapid diagnostic test (RDT) or microscopy

Appendix H: Relative difference in reactive case detection (RACD) positivity by LAMP/PCR versus RDT/microscopy. Summary estimates are reported by transmission strata and generated using a random effects model.

**Appendix A: Literature search terms and exclusion criteria**

Search terms: malaria AND reactive case detection, OR active case detection, OR proactive case detection, OR mass screen and treat, OR MSAT, OR mass test and treat, OR MTAT, OR focal screen and treat, OR FSAT, OR focal test and treat, OR FTAT, OR case investigation, OR reactive case investigation, OR case follow up, OR contact tracing, OR test treat and track, OR elimination case finding, OR elimination blood survey, OR elimination surveillance.

Exclusion criteria:

1. Studies on diseases other than malaria
2. Studies focused on the immunology, entomology, ecology, or genetics of malaria
3. Cross-sectional surveys designed to establish prevalence or characterize transmission patterns in a population
4. Studies of community case management of fever
5. TaT studies that target only pregnant women, infants, or school children
6. TaT studies that do not provide information on the treatment component
7. Studies that focus solely on qualitative aspects of a TaT intervention (e.g., community perception and acceptance of TaT, costs of TaT, programmatic perspectives on TaT) and do not provide quantitative details
8. TaT study protocols
9. Non-English language studies

**Appendix B: WHO categories of malaria transmission intensity**^[[1]](#footnote-1)^

**High transmission**: annual parasite incidence of 450 or more cases per 1000 population and a *P. falciparum* prevalence rate of ≥35%.

**Moderate transmission**: annual parasite incidence of 250−450 cases per 1000 population and a *P. falciparum*/*P. vivax* prevalence rate of 10−35%.

**Low transmission**: annual parasite incidence of 100−250 cases per 1000 population and a *P. falciparum*/*P. vivax* prevalence rate of 1−10%.

**Very low transmission**: annual parasite incidence of <100 cases per 1000 population and a *P. falciparum/P. vivax* prevalence rate of >0 but <1%.

**Appendix C: Studies assessed for inclusion (n=235)**

The 148 excluded studies are in *italics* and the 87 included studies are in **bold** (note that 8 of these studies were subdivided, resulting in 96 total studies included in the analysis).

1. *Abeyasinghe RR, Galappaththy GNL, Smith Gueye C, Kahn JG, Feachem RGA. Malaria control and elimination in Sri Lanka: documenting progress and success factors in a conflict setting. PLoS ONE 2012; 7(8): e43162.*
2. **Aidoo EK, Afrane YA, Machani MG, Chebore W, Lawson BW, et al. Reactive case detection of *Plasmodium falciparum* in western Kenya highlands: effective in identifying additional cases, yet limited effect on transmission. *Malar J* 2018; 17:111.**
3. *Atkinson JA, Johnson ML, Wijesinghe R, Bobogare A, Losi L, et al. Operational research to inform a sub-national surveillance intervention for malaria elimination in Solomon Islands. Malar J 2012; 11:101.*
4. *Aung PP, Thein ZW, Hein ZN, Aung KT, Mon NO, et al. Challenges in early phase of implementing the 1-3-7 surveillance and response approach in malaria elimination setting: a field study from Myanmar. Infect Dis Poverty 2020; 9(1): 1-3.*
5. *Aydin-Schmidt B, Xu W, Gonzalez IJ, Polley SD, Bell D, et al. Loop mediated isothermal amplification (LAMP) accurately detects malaria DNA from filter paper blood samples of low density parasitaemias. PLoS ONE 2014; 9(8): e103905.*
6. *Aydin-Schmidt B, Morris U, Ding XC, Jovel I, Msellem MI, et al. Field evaluation of a high throughput loop mediated isothermal amplification test for the detection of asymptomatic Plasmodium infections in Zanzibar. PLoS ONE 2017; 12(1) e0169037.*
7. *Bal M, Das A, Ghosal J, Pradhan MM, Khuntia HK, et al. Assessment of effectiveness of DAMaN: a malaria intervention program initiated by Government of Odisha, India. PLoS ONE 2020; 15(9): e0238323.*
8. *Baltzell KA, Shakely D, Hsiang M, Kemere J, Ali AS, et al. Prevalence of PCR detectable malaria infection among febrile patients with a negative Plasmodium falciparum specific rapid diagnostic test in Zanzibar. Am J Trop Med Hyg 2013; 88(2): 289-291.*
9. **Bansil P, Yeshiwondim AK, Guinovart C, Serda B, Scott C, et al. Malaria case investigation with reactive focal testing and treatment: operational feasibility and lessons learned from low and moderate transmission areas in Amhara Region, Ethiopia. *Malar J* 2018; 17:449.**
10. *Barbosa S, Gozze AB, Lima NF, Batista CL, da Silva Bastos M, et al. Epidemiology of disappearing Plasmodium vivax malaria: a case study in rural Amazonia. PLoS Negl Trop Dis 2014; 8(8): e3109.*
11. *Baum E, Sattabongkot J, Sirichaisinthop J, Kiattibutr K, Jain A, et al. Common asymptomatic and submicroscopic malaria infections in Western Thailand revealed in longitudinal molecular and serological studies: a challenge to malaria elimination. Malar J 2016; 15:333.*
12. **Bekolo CE, Williams TD. Adding proactive and reactive case detection into the integrated community case management system to optimize diagnosis and treatment of malaria in a high transmission setting of Cameroon: an observational quality improvement study. BMJ Open 2019; 9:e026678.**
13. *Bell D, Fleurent AE, Hegg MC, Boomgard JD, McConnico CC. Development of new malaria diagnostics: matching performance and need. Malar J 2016; 15:406.*
14. **Bharti PK, Rajvanshi H, Nisar S, Jayswar H, Saha KB, et al. Demonstration of indigenous malaria elimination through Track-Test-Treat-Track (T4) strategy in a Malaria Elimination Demonstration Project in Mandla, Madhya Pradesh. *Malar J* 2020; 19:339.**
15. *Bhondoekhan FR, Searle KM, Hamapumbu H, Lubinda M, Matoba J, et al. Improving the efficiency of reactive case detection for malaria elimination in southern Zambia: a cross-sectional study. Malar J 2020; 19:175.*
16. **Bjorkman A, Cook J, Sturrock H, Msellem M, Ali A, et al. Spatial distribution of falciparum malaria infections in Zanzibar: implications for focal drug administration strategies targeting asymptomatic parasite carriers. *Clin Infect Dis* 2017; 64(9): 1236-1243.**
17. **Bousema T, Stresman G, Baidjoe AY, Bradley J, Knight P, et al. The impact of hotspot-targeted interventions on malaria transmission in Rachuonyo South District in the Western Kenyan highlands: a cluster-randomized controlled trial. *PLoS Med* 2016; 13(4): e1001993.**
18. *Bousema T, Okell L, Felger I, Drakeley C. Asymptomatic malaria infections: detectability, transmissibility and public health relevance. Nat Rev Microbiol 2014; 12(1): 833-840.*
19. *Bousema T, Griffin JT, Sauerwein RW, Smith DL, Churcher TS, et al. Hitting hotspots: spatial targeting of malaria for control and elimination. PLoS Med 2012; 9(1): e1001165.*
20. **Branch O, Casapia WM, Gamboa DV, Hernandez JN, Alava FF, et al. Clustered local transmission and asymptomatic *Plasmodium falciparum* and *Plasmodium vivax* malaria infections in a recently emerged, hypoendemic Peruvian Amazon community. *Malar J* 2005; 4:27.**
21. **Bridges DJ, Chishimba S, Mwenda M, Winters AM, Slawsky E, et al. The use of spatial and genetic tools to assess *Plasmodium falciparum* transmission in Lusaka, Zambia between 2011 and 2015. *Malar J* 2020; 19:20.**
22. *Bridges DJ, Miller JM, Chalwe V, Moonga H, Hamainza B, et al. Community-led responses for elimination (CoRE): a study protocol for a community randomized controlled trial assessing the effectiveness of community-level, reactive focal drug administration for reducing Plasmodium falciparum infection prevalence and incidence in Southern Province, Zambia. Trials 2017; 18:511.*
23. *Campillo A, Daily J, Gonzalez IJ. International survey to identify diagnostic needs to support malaria elimination: guiding the development of combination highly sensitive rapid diagnostic tests. Malar J 2017; 16:385.*
24. *Canier L, Khim N, Kim S, Sluydts V, Heng S, et al. An innovative tool for moving malaria PCR detection of parasite reservoir into the field. Malar J 2013; 12:405.*
25. *Cao J, Sturrock HJW, Cotter C, Zhou S, Zhou H, et al. Communicating and monitoring surveillance and response activities for malaria elimination: China’s “1-3-7” strategy. PLoS Med 2014; 11(5): e1001642.*
26. *Chen SC, Chang HL, Chen KT. The epidemiology of imported malaria in Taiwan between 2002-2013: the importance of sensitive surveillance and implications for pre-travel medical advice. Int J Environ Res Public Health 2014; 11(6): 5651-5664.*
27. **Chihanga S, Haque U, Chanda E, Mosweunyane T, Moakofhi K, et al. Malaria elimination in Botswna, 2012-2014: achievements and challenges. *Parasit Vectors* 2016; 9:99.**
28. **Chitnis N, Pemberton-Ross P, Yukich J, Hamainza B, Miller J, et al. Theory of reactive interventions in the elimination and control of malaria. *Malar J* 2019; 18:266.**
29. *Chourasia MK, Raghavendra K, Bhatt RM, Swain DK, Valecha N, Kleinschmidt I. Burden of asymptomatic malaria among a tribal population in a forested village of central India: a hidden challenge for malaria control in India. R Soc Pub Health 2017; 147: 92-97.*
30. *Cohen R, Cardona JS, Navarro ES, Padilla N, Reyes L, et al. Outbreak investigation of Plasmodium vivax malaria in a region of Guatemala targeted for malaria elimination. Am J Trop Med Hyg 2017; 96(4) 819-825.*
31. *Collins KA, Ouedraogo A, Guelbeogo WM, Awandu SS, Stone W, et al. Investigating the impact of enhanced community case management and monthly screening and treatment on the transmissibility of malaria infections in Burkina Faso: study protocol for a cluster-randomised trial. BMJ Open 2019; 9: e030598.*
32. *Congpuong K, SaeJeng A, Sug-aram R, Aruncharus S, Darakapong A, et al. Mass blood survey for malaria: pooling and real-time PCR combined with expert microscopy in north-west Thailand. Malar J 2012; 11:288.*
33. **Conner RO, Dieye Y, Hainsworth M, Tall A, Cisse B, et al. Mass testing and treatment for malaria followed by weekly fever screening, testing and treatment in Northern Senegal: feasibility, cost and impact. *Malar J* 2020; 19:252.**
34. **Cook J, Xu W, Msellem M, Vonk M, Bergstrom B, et al. Mass screening and treatment on the basis of results of a *Plasmodium falciparum*-specific rapid diagnostic test did not reduce malaria incidence in Zanzibar. *J Infect Dis* 2014; 211(9): 1476-1483.**
35. **Cook J, Aydin-Schmidt B, Gonzalez IJ, Bell D, Edlund E, et al. Loop-mediated isothermal amplification (LAMP) for point-of-care detection of asymptomatic low-density malaria parasite carriers in Zanzibar. *Malar J* 2015; 14:43.**
36. **Cotter C, Sudathip P, Herdiana H, Cao Y, Liu Y, et al. Piloting a programme tool to evaluate malaria case investigation and reactive case detection activities: results from 3 settings in the Asia Pacific. *Malar J* 2017; 16:347.**
37. *Cotter C, Sturrock HJW, Hsiang MS, Liu J, Phillips AA, et al. The changing epidemiology of malaria elimination: new strategies for new challenges. Lancet 2013; 382(9895): 900-911.*
38. **Crowell V, Brief OJT, Hardy D, Chitnis N, Maire N, et al. Modeling the cost-effectiveness of mass screening and treatment for reducing *Plasmodium falciparum* malaria burden. *Malar J* 2012; 11(Suppl 1): P19.**
39. *de AW Gunasekera WMKT, Premaratne R, Fernando D, Munaz M, Piyasena MG, et al. A comparative analysis of the outcome of malaria case surveillance strategies in Sri Lanka in the prevention of re-establishment phase. Malar J 2021; 20:80.*
40. *de AW Gunasekera WMKT, Abeyasinghe RR, Premawansa S, Fernando SD. Usefulness of polymerase chain reaction to supplement field microscopy in a pre-selected population with a high probability of malaria infections. Am J Trop Med Hyg 2011; 85(1): 6-11.*
41. *Deen J, Mukaka M, von Seidlein L. What is the yield of malaria reactive case detection in the Greater Mekong Sub-region? A review of published data and meta-analysis. Malar J 2021; 20:131.*
42. **Desai MR, Samuels AM, Odongo W, Williamson J, Odero NA, et al. Impact of intermittent mass testing and treatment on incidence of malaria infection in a high transmission area of western Kenya. *Am J Trop Med Hyg* 2020; 103(1): 369.**
43. **Deutsch-Feldman M, Hamapumbu H, Lubinda J, Musonda M, Katowa B, et al. Efficiency of a malaria-reactive test-and-treat program in southern Zambia: a prospective, observational study. *Am J Trop Med Hyg* 2018; 98(5): 1382-1388.**
44. *Dharmawardena P, Premaratne R, Wickremasinghe R, Mendis K, Fernando D. Epidemiological profile of imported malaria cases in the prevention of reestablishment phase in Sri Lanka. Path Glob Health 2021; 1-9.*
45. *Ding XC, Ade MP, Baird JK, Cheng Q, Cunningham J, et al. Defining the next generation of Plasmodium vivax diagnostic tests for control and elimination: target product profiles. PLoS Negl Trop Dis 2017; 11(4): e0005516.*
46. **Donald W, Pasay C, Guintran JO, Iata H, Anderson K, et al. The utility of malaria rapid diagnostic tests as a tool in enhanced surveillance for malaria elimination in Vanuatu. *PLoS ONE* 2016; 11(11): e0167136.**
47. *Dondorp AM, Smithius FM, Woodrow C, von Seidlein L. How to contain artemisinin- and multidrug-resistant falciparum malaria. Trends Parasitol 2017; 33(5): 353-363.*
48. **Eisele TP, Bennett A, Silumbe K, Finn TP, Chalwe V, et al. Short-term impact of mass drug administration with dihydroartemisinin plus piperaquine on malaria in Southern Province Zambia: a cluster-randomized controlled trial. *J Infect Dis* 2016; 214(12): 1831-1839.**
49. *Ekawati LL, Johnson KC, Jacobson JO, Cueto CA, Zarlinda I, et al. Defining malaria risks among forest workers in Aceh, Indonesia: a formative assessment. Malar J 2020; 19:441.*
50. *Ekawati LL, Herdiana H, Sumiwi ME, Barussanah C, Ainun C, et al. A comprehensive assessment of the malaria microscopy system of Aceh, Indonesia, in preparation for malaria elimination. Malar J 2015; 14:240.*
51. *Elbadry MA, Al-Khedery B, Tagliamonte MS, Yowell CA, Raccurt CP, et al. High prevalence of asymptomatic malaria infections: a cross-sectional study in rural areas in six departments in Haiti. Malar J 2015; 14:510.*
52. *Elliott RC, Smith DL, Echodu DC. Synergy and timing: a concurrent mass medical campaign predicted to augment indoor residual spraying for malaria. Malar J 2019; 18:160.*
53. *Faye S, Cico A, Gueye AB, Baruwa E, Johns B, Ndiop M, Alilio M. Scaling up malaria intervention “packages” in Senegal: using cost effectiveness data for improving allocative efficiency and programmatic decision-making. Malar J 2018; 17:159.*
54. **Feng J, Tu H, Zhang L, Zhang S, Jiang S, Xia Z, Zhou S. Mapping transmission foci to eliminate malaria in the People’s Republic of China, 2010-2015: a retrospective analysis. *BMC Infect Dis* 2018; 18(1): 115.**
55. *Feng J, Liu J, Feng X, Zhang L, Xiao H, Xia Z. Towards malaria elimination: monitoring and evaluation of the “1-3-7” approach at the China-Myanmar border. Am J Trop Med Hyg 2016; 95(4): 806-810.*
56. *Fernando SD, Ainan S, Premaratne RG, Rodrigo C, Jayanetti SR, Rajapakse S. Challenges to malaria surveillance following elimination of indigenous transmission: findings from a hospital-based study in rural Sri Lanka. Int Health 2015; 7(5): 317-323.*
57. **Fontoura PS, Finco BF, Lima NF, de Carvalho Jr JF, Vinetz JM, et al. Reactive case detection for *Plasmodium vivax* malaria elimination in rural Amazonia. *PLoS Negl Trop Dis* 2016; 10(12): e0005221.**
58. *Galactinova K, Velarde M, Silumbe K, Miller J, McDonnell A, et al. Costing malaria interventions from pilots to elimination programmes. Malar J 2020; 19:332.*
59. **Gerardin J, Bever CA, Bridenbecker D, Hamainza B, Silumbe K, et al. Effectiveness of reactive case detection for malaria elimination in three archetypical transmission settings: a modelling study. *Malar J* 2017; 16:248.**
60. **Gerardin J, Bever CA, Hamainza B, Miller JM, Eckhoff PA, Wenger EA. Optimal population-level infection detection strategies for malaria control and elimination in a spatial model of malaria transmission. *PLoS Comput Biol* 2016; 12(1): e1004707.**
61. **Gerardin J, Ouedraogo AL, McCarthy KA, Eckhoff PA, Wenger EA. Characterization of the infectious reservoir of malaria with an agent-based model calibrated to age-stratified parasite densities and infectiousness. *Malar J* 2015; 14:231.**
62. **Gerardin J, Eckhoff P, Wenger EA. Mass campaigns with antimalarial drugs: a modelling comparison of artemether-lumefantrine and DHA-piperaquine with and without primaquine as tools for malaria control and elimination. *BMC Infect Dis* 2015; 15:144.**
63. *Gosling RD, Okell L, Mosha J, Chandramohan D. The role of antimalarial treatment in the elimination of malaria. Clin Microbiol Infect 2011; 17(11): 1617-1623.*
64. *Grietens KP, Gryseels C, Dierickx S, Bannister-Tyrrell M, Trienekens S, et al. Characterizing types of human mobility to inform differential and targeted malaria elimination strategies in northeast Cambodia. Sci Rep 2015; 5:15837.*
65. **Griffin JT, Hollingsworth TD, Okell LC, Churcher TS, White M, et al. Reducing *Plasmodium falciparum* malaria transmission in Africa: a model-based evaluation of intervention strategies. *PLoS Med* 2010; 7(8): e1000324.**
66. *Hamainza B, Moonga H, Sikaala CH, Kamuliwo M, Bennett A, et al. Monitoring, characterization and control of chronic, symptomatic malaria infections in rural Zambia through monthly household visits by paid community health workers. Malar J 2014; 13:128.*
67. **Hamze H, Charchuk R, Jean Paul MK, Claude KM, Leon M, Hawkes MT. Lack of household clustering of malaria in a complex humanitarian emergency: implications for active case detection. *Pathog Glob Health* 2016; 110(6): 223-227.**
68. *Harris I, Sharrock WW, Bain LM, Gray KA, Bobogare A, et al. A large proportion of asymptomatic Plasmodium infections with low and sub-microscopic parasite densities in the low transmission setting of Temotu Province, Solomon Islands: challenges for malaria diagnostics in an elimination setting. Malar J 2010; 9:254.*
69. **Herdiana H, Cotter C, Coutrier FN, Zarlinda I, Zelman BW, et al. Malaria risk factor assessment using active and passive surveillance data from Aceh Besar, Indonesia, a low endemic, malaria elimination setting with *Plasmodium knowlesi*, *Plasmodium vivax*, and *Plasmodium falciparum*. *Malar J* 2016; 15:468.**
70. *Herdiana H, Fuad A, Asih PBS, Zubaedah S, Arisanti RR, et al. Progress towards malaria elimination in Sabang Municipality, Aceh, Indonesia. Malar J 2013; 12:42.*
71. **Herdiana H, Irnawati I, Coutrier FN, Munthe A, Mardiati M, et al. Two clusters of *Plasmodium knowlesi* cases in a malaria elimination area, Sabang Municipality, Aceh, Indonesia. *Malar J* 2018; 17:186.**
72. *Hiwat H, Hardjopawiro LS, Takken W, Villegas L. Novel strategies lead to pre-elimination of malaria in previously high-risk areas in Suriname, South America. Malar J 2012; 11:10.*
73. **Hoyer S, Nguon S, Kim S, Habib N, Khim N, et al. Focused screening and treatment (FSAT): a PCR-based strategy to detect malaria parasite carriers and contain drug resistant *P. falciparum*, Pailin, Cambodia. *PLoS ONE* 2012; 7(10): e45797.**
74. **Hsiang MS, Ntshalintshali N, Dufour MK, Dlamini N, Nhlabathi N, et al. Active case finding for malaria: a 3-year national evaluation of optimal approaches to detect infections and hotspots through reactive case detection in the low-transmission setting of Eswatini. *Clin Infect Dis* 2019.**
75. **Hsiang MS, Ntuku H, Roberts KW, Dufour MS, Whittemore B, et al. Effectiveness of reactive focal mass drug administration and reactive focal vector control to reduce malaria transmission in the low malaria-endemic setting of Namibia: a cluster-randomised controlled, open-label, two-by-two factorial design trial. *Lancet* 2020; 395(10233): 1361-1373.**
76. *Hsiang MS, Greenhouse B, Rosenthal PJ. Point of care testing for malaria using LAMP, loop mediated isothermal amplification. J Infect Dis 2014; 210(8): 1167-1169.*
77. *Hsiang MS, Hwang J, Kunene S, Drakeley C, Kandula D, et al. Surveillance for malaria elimination in Swaziland: a national cross-sectional study using pooled PCR and serology. PLoS ONE 2012; 7(1): e29550.*
78. **Hustedt J, Canavati SE, Rang C, Ashton RA, Khim N, et al. Reactive case-detection of malaria in Pailin Province, Western Cambodia: lessons from a year-long evaluation in a pre-elimination setting. *Malar J* 2016; 15:132.**
79. *Jaiteh F, Okebe J, Masunaga Y, D’Alessandro U, Achan J, et al. Understanding adherence to reactive treatment of asymptomatic malaria infections in The Gambia. Sci Rep 2021; 11:1746.*
80. *Jeffree SM, Ahmed K, Safian N, Hassan R, Mihat O, et al. Falciparum malaria outbreak in Sabah linked to an immigrant rubber tapper. Am J Trop Med Hyg 2018; 98(1): 45-50.*
81. *Kalantari M, Soltani Z, Ebrahimi M, Yousefi M, Amin M, et al. Monitoring of Plasmodium infection in humans and potential vectors of malaria in a newly emerged focus in southern Iran. Pathog Glob Health 2017; 111(1): 49-55.*
82. **Karunasena VM, Marasinghe M, Koo C, Amarasinghe S, Senaratne AS, et al. The first introduced malaria case reported from Sri Lanka after elimination: implications for preventing the re-introduction of malaria in recently eliminated countries. *Malar J* 2019; 18:210.**
83. **Kern SE, Tiono AB, Makanga M, Gbadoe AD, Premji Z, et al. Community screening and treatment of asymptomatic carriers of *Plasmodium falciparum* with artemether-lumefantrine to reduce malaria disease burden: a modelling and simulation analysis. *Malar J* 2011; 10:210.**
84. *Khandekar E, Kramer R, Ali AS, Al-Mafazy A-W, Egger JR, et al. Evaluating response time in Zanzibar’s malaria elimination case-based surveillance-response system. Am J Trop Med Hyg 2019; 100(2):256-263.*
85. **Kheang ST, Lin MA, Lwin S, Naing YH, Yarzar P, Kak N, Price T. Malaria case detection among mobile populations and migrant workers in Myanmar: comparisons of 3 service delivery approaches. *Glob Health Sci Pract* 2018; 6(2):384-389.**
86. **Kheang ST, Sovannaroth S, Barat LM, Dysoley L, Kapella BK, et al. Malaria elimination using the 1-3-7 approach: lessons from Sampov Loun, Cambodia. *BMC Pub Health* 2020; 20(1): 1-7.**
87. *Kim S, Luande VN, Rocklov J, Carlton JM, Tozan Y. A systematic review of the evidence on the effectiveness and cost-effectiveness of mass screen-and-treat interventions for malaria control. Am J Trop Med Hyg 2021; 105(6): 1722.*
88. *Koita K, Novotny J, Kunene S, Zulu Z, Ntshalintshali N, Gandhi M, Gosling R. Targeting imported malaria through social networks: a potential strategy for malaria elimination in Swaziland. Malar J 2013; 12:219.*
89. *Kosasih A, Koepfli C, Dahlan MS, Hawley WA, Baird JK, et al. Gametocyte carriage of Plasmodium falciparum (pfs25) and Plasmodium vivax (pvs25) during mass screening and treatment in West Timor, Indonesia: a longitudinal prospective study. Malar J 2021; 20:177.*
90. **Kunkel A, Nguon C, Iv S, Chhim S, Peov D, et al. Choosing interventions to eliminate forest malaria: preliminary results of two operational research studies inside Cambodian forests. *Malar J* 2021; 20:51.**
91. *Kyaw AMM, Kathirvel S, Das M, Thapa B, Linn NYY, et al. “Alert-Audit-Act”: assessment of surveillance and response strategy for malaria elimination in three low-endemic settings of Myanmar in 2016. Trop Med Health 2018; 46:11.*
92. *Landier J, Parker DM, Thu AM, Lwin KM, Delmas G, et al. Effect of generalized access to early diagnosis and treatment and targeted mass drug administration on Plasmodium falciparum malaria in Eastern Myanmar: an observational study of a regional elimination programme. Lancet 2018; 391:1916-1926.*
93. *Landier J, Parker DM, Thu AM, Carrara VI, Lwin KM, et al. The role of early detection and treatment in malaria elimination. Malar J 2016; 15:363.*
94. *Larsen DA, Winters A, Cheelo S, Hamainza B, Kamuliwo M, Miller JM, Bridges DJ. Shifting the burden or expanding access to care? Assessing malaria trends following scale-up of community health worker malaria case management and reactive case detection. Malar J 2017; 16:441.*
95. **Larsen DA, Ngwenya-Kangombe T, Cheelo S, Hamainza B, Miller J, et al. Location, location, location: environmental factors better predict malaria-positive individuals during reactive case detection than index case demographics in Southern Province, Zambia. *Malar J* 2017; 16:18.**
96. **Larsen DA, Chisha Z, Winters B, Mwanza M, Kamuliwo M, et al. Malaria surveillance in low-transmission areas of Zambia using reactive case detection. *Malar J* 2015; 14:465.**
97. **Larsen DA, Bennett A, Silumbe K, Hamainza B, Yukich JO, et al. Population-wide malaria testing and treatment with rapid diagnostic tests and artemether-lumefantrine in Southern Zambia: a community randomized step-wedge control trial design. *Am J Trop Med Hyg* 2015; 92(5): 913-921.**
98. *Larson BA, Ngoma T, Silumbe K, Rutagwera MI, Hamainza B, et al. A framework for evaluating the costs of malaria elimination interventions: an application to reactive case detection in Southern Province of Zambia, 2014. Malar J 2016; 15:408.*
99. **Lee PW, Liu CT, Rampao HS, do Rosario VE, Shaio MF. Pre-elimination of malaria on the island of Principe. *Malar J* 2010; 9:26.**
100. *Lek D, Popovici J, Ariey F, Vinjamuri SB, Meek S, et al. National malaria prevalence in Cambodia: microscopy versus polymerase chain reaction estimates. Am J Trop Med Hyg 2016; 95(3): 588-594.*
101. *Lek D, Callery JJ, Nguon C, Debackere M, Sovannaroth S, et al. Tools to accelerate falciparum malaria elimination in Cambodia: a meeting report. Malar J 2020; 19:151.*
102. *Lennon SE, Miranda A, Henao J, Vallejo AF, Perez J, et al. Malaria elimination challenges in Mesoamerica: evidence of submicroscopic malaria reservoirs in Guatemala. Malar J 2016: 15:441.*
103. *Lertpiriyasuwat C, Sudathip P, Kitchakarn S, Areechokchai D, Naowarat S, et al. Implementation and success factors from Thailand’s 1-3-7 surveillance strategy for malaria elimination. Malar J 2021; 20:201.*
104. *Liew JWK, binti Mahpot R, Dzul S, bin Abdul Razak HA, binti Ahmad Shah Azizi NA, et al. Importance of proactive malaria case surveillance and management in Malaysia. Am J Trop Med Hyg 2018; 98(6):1709-1713.*
105. *Lin JT, Saunders DL, Meshnick SR. The role of submicroscopic parasitemia in malaria transmission: what is the evidence? Trends Parasitol 2014; 30(4): 183-190.*
106. *Lindblade KA, Steinhardt L, Samuels A, Kachur SP, Slutsker L. The silent threat: asymptomatic parasitemia and malaria transmission. Expert Rev Anti Infect Ther 2013; 11(6): 623-639.*
107. *Linn AM, Ndiaye Y, Hennessee I, Gaye S, Linn P, et al. Reduction in symptomatic malaria prevalence through proactive community treatment in rural Senegal. Trop Med Int Health 2015; 20(11): 1438-1446.*
108. **Littrell M, Sow GD, Ngom A, Ba M, Mboup BM, et al. Case investigation and reactive case detection for malaria elimination in northern Senegal. *Malar J* 2013; 12:331.**
109. *Lohfeld L, Kangombe-Ngwenya T, Winters AM, Chisha Z, Hamainza B, et al. A qualitative review of implementer perceptions of the national community-level malaria surveillance system in Southern Province, Zambia. Malar J 2016; 15:400.*
110. *Lourenco C, Kandula D, Haidula L, Ward A, Cohen JM. Strengthenin malaria diagnosis and appropriate treatment in Namibia: a test of case management training interventions in Kavango Region. Malar J 2014; 13:508.*
111. *Lover AA, Dantzer E, Hocini S, Estera R, Rerolle F, et al. Study protocol for a cluster-randomized split-plot design trial to assess the effectiveness of targeted active malaria case detection among high-risk populations in Southern Lao PDR (the AcME-Lao study). Gates Open Res 2019; 3.*
112. *Lu G, Liu Y, Beiersmann C, Feng Y, Cao J, Muller O. Challenges in and lessons learned during the implementation of the 1-3-7 malaria surveillance and response strategy in China: a qualitative study. Infect Dis Poverty 2016; 5:94.*
113. *Ma S, Lawpoolsri S, Soonthornworasiri N, Khamsiriwatchara A, Jandee K, et al. Effectiveness of implementation of electronic malaria information system as the national malaria surveillance system in Thailand. JMIR Public Health Surveill 2016; 2(1): e20.*
114. *Macauley C. Aggressive active case detection: a malaria control strategy based on the Brazilian model. Soc Sci Med 2005; 60(3): 563-573.*
115. *malERA Consultative Group on Monitoring, Evaluation, and Surveillance. A research agenda for malaria eradication: monitoring, evaluation, and surveillance. PLoS Med 2011; 8(1): e1000400.*
116. *Manjurano A, Okell L, Lukindo T, Reyburn H, Olomi R, et al. Association of sub-microscopic malaria parasite carriage with transmission intensity in north-eastern Tanzania. Malar J 2011; 10:370.*
117. *Manning J, Lon C, Spring M, Wojnarski M, Somethy S, et al. Cluster-randomized trial of monthly malaria prophylaxis versus focused screening and treatment: a study protocol to define malaria elimination strategies in Cambodia. Trials 2018; 19:558.*
118. *Marasinghe MM, Karunasena VM, Seneratne AS, Herath HD, Fernando D, et al. Mass radical treatment of a group of foreign workers to mitigate the risk of re-establishment of malaria in Sri Lanka. Malar J 2020; 19:346.*
119. *Marshall JM, Bennett A, Kiware SS, Sturrock HJW. The hitchhiking parasite: why human movement matters to malaria transmission and what we can do about it. Trends Parasitol 2016; 32(10): 752-755.*
120. *Medzihradsky OF, Kleinschmidt I, Mumbengegwi D, Roberts KW, McCreesh P, et al. Study protocol for a cluster randomized controlled factorial design trial to assess the effectiveness and feasibility of reactive focal mass drug administration and vector control to reduce malaria transmission in the low endemic setting of Namibia. BMJ Open 2018; 8:e019294.*
121. **Meredith HR, Wesolowski A, Menya D, Esimit D, Lokoel G, et al. Epidemiology of *Plasmodium falciparum* infections in a semi-arid rural African setting: evidence of reactive case detection in Northwestern Kenya. *Am J Trop Med Hyg* 2021; 105(4): 1076-1084.**
122. *Millar J, Toh KB, Valle D. To screen or not to screen: an interactive framework for comparing costs of mass malaria treatment interventions. BMC Med 2020; 18(1): 1-14.*
123. **Mlacha YP, Wang D, Chaki PP, Gavana T, Zhou Z, et al. Effectiveness of the innovative 1, 7-malaria reactive community-based testing and response (1, 7-mRCTR) approach on malaria burden reduction in Southeastern Tanzania. *Malar J* 2020; 19:292.**
124. **Molina Gomez K, Caicedo MA, Gaitan A, Herrera-Varela M, Arce MI, et al. Characterizing the malaria rural-to-urban transmission interface: the importance of reactive case detection. *PLoS Negl Trop Dis* 2017; 11(7): e0005780.**
125. *Montenegro CC, Bustamante-Chauca TP, Reyes CP, Bernal M, Gonzales L, et al. Plasmodium falciparum outbreak in native communities of Condorcanqui, Amazonas, Peru. Malar J 2021; 20:88.*
126. *Moonen B, Cohen JM, Snow RW, Slutsker L, Drakeley C, et al. Operational strategies to achieve and maintain malaria elimination. Lancet 2010; 376(9752): 1592-1603.*
127. *Morales DO, Quinatoa PA, Cagua JC. Characterization of an outbreak of malaria in a non-endemic zone on the coastal region of Ecuador. Biomedica 2021; 41(Suppl 1): 100-112.*
128. **Moreno-Gutierrez D, Llanos-Cuentas A, Barboza JL, Contreras-Mancilla J, Gamboa D, et al. Effectiveness of a malaria surveillance strategy based on active case detection during high transmission season in the Peruvian Amazon. *Int J Environ Res Public Health* 2018; 15(12):2670.**
129. *Morris U, Khamis M, Aydin-Schmidt B, Abass AK, Msellem MI, et al. Field deployment of loop-mediated isothermal amplification for centralized mass-screening of asymptomatic malaria in Zanzibar: a pre-elimination setting. Malar J 2015; 14:205.*
130. **Mosha JF, Sturrock HJW, Greenhouse B, Greenwood B, Sutherland CJ, et al. Epidemiology of subpatent *Plasmodium falciparum* infection: implications for detection of hotspots with imperfect diagnostics. *Malar J* 2013; 12:221.**
131. *Motshoge T, Ababio GK, Aleksenko L, Read J, Peloewetse E, et al. Molecular evidence of high rates of asymptomatic P. vivax infection and very low P. falciparum malaria in Botswana. BMC Infect Dis 2016; 16:520.*
132. *Mukaka M, Peerawaranun P, Parker DM, Kajeechiwa L, Nosten FH, et al. Clustering of malaria in households in the Greater Mekong Subregion: operational implications for reactive case detection. Malar J 2021; 20:351.*
133. **Mwesigwa J, Slater H, Bradley J, Saidy B, Ceesay F, et al. Field performance of the malaria highly sensitive rapid diagnostic test in a setting of varying malaria transmission. *Malar J* 2019; 18:288.**
134. *Mwingira F, Genton B, Kabanywanyi ANM, Felger I. Comparison of detection methods to estimate asexual Plasmodium falciparum parasite prevalence and gametocyte carriage in a community survey in Tanzania. Malar J 2014; 13:433.*
135. **Ndong IC, Okyere D, Enos JY, Mensah BA, Nyarko A, et al. Prevalence of asymptomatic malaria parasitaemia following mass testing and treatment in Pakro sub-district of Ghana. *BMC Pub Health* 2019; 19:1622.**
136. *Ndong IC, Okyere D, Enos JY, Amambua-Ngwa A, Merle CSC, et al. Challenges and perceptions of implementing mass testing, treatment and tracking in malaria control: a qualitative study in Pakro sub-district of Ghana. BMC Pub Health 2019; 19:695.*
137. *Nikolov M, Bever CA, Upfill-Brown A, Hamainza B, Miller JM, et al. Malaria elimination campaigns in the Lake Kariba region of Zambia: a spatial dynamical model. PLoS Comput Biol 2016; 12(11): e1005192.*
138. *Noor AM, Mohamed MB, Mugyenyi CK, Osman MA, Guessod HH, et al. Establishing the extent of malaria transmission and challenges facing pre-elimination in the Republic of Djibouti. BMC Infect Dis 2011; 11:121.*
139. *Nourein AB, Abass MA, Nugud AHD, El Hassan I, Snow RW, Noor AM. Identifying residual foci of Plasmodium falciparum infections for malaria elimination: the urban context of Khartoum, Sudan. PLoS ONE 2011; 6(2): e16948.*
140. *Nwe TW, Oo T, Wai KT, Zhou S, van Griensven J, et al. Malaria profiles and challenges in artemisinin resistance containment in Myanmar. Infect Dis Poverty 2017; 6:76.*
141. *Nygren D, Isaksson AL. Battling malaria in rural Zambia with modern technology: a qualitative study on the value of cell phones, geographical information systems, asymptomatic carriers and rapid diagnostic tests to identify, treat and control malaria. J Public Health Afr 2014; 5(1): 171.*
142. *O’Sullivan M, Kenilorea G, Yamaguchi Y, Bobogare A, Losi L, et al. Malaria elimination in Isabel Province, Solomon Islands: establishing a surveillance-response system to prevent introduction and reintroduction of malaria. Malar J 2011; 10:235.*
143. *Odero NA, Samuels AM, Odongo W, Abong’o B, Gimnig J, et al. Community-based intermittent mass testing and treatment for malaria in an area of high transmission intensity, western Kenya: development of study site infrastructure and lessons learned. Malar J 2019; 18:255.*
144. *Okebe J, Ribera JM, Balen J, Jaiteh F, Masunaga Y, et al. Reactive community-based self-administered treatment against residual malaria transmission: study protocol for a randomized controlled trial. Trials 2018; 19:126.*
145. *Okell LC, Bousema T, Griffin JT, Ouedraogo AL, Ghani AC, Drakeley CJ. Factors determining the occurrence of submicroscopic malaria infections and their relevance for control. Nat Commun 2012; 3:1237.*
146. **Parker DM, Landier J, von Seidlein L, Dondorp A, White L, et al. Limitations of malaria reactive case detection in an area of low and unstable transmission on the Myanmar-Thailand border. *Malar J* 2016; 15:571.**
147. *Parker DM, Matthews SA, Yan G, Zhou G, Lee MC, et al. Microgeography and molecular epidemiology of malaria at the Thailand-Myanmar border in the malaria pre-elimination phase. Malar J 2015; 14:198.*
148. *Perera R, Caldera A, Wickremasinghe AR. Reactive case detection (RACD) and foci investigation strategies in malaria control and elimination: a review. Malar J 2020; 19:401.*
149. *Phommasone K, Adhikari B, Henriques G, Pongvongsa T, Phongmany P, et al. Asymptomatic Plasmodium infections in 18 villages of southern Savannakhet Province, Lao PDR (Laos). Malar J 2016; 15:296.*
150. **Pinchoff J, Henostroza G, Carter BS, Roberts ST, Hatwiinda S, et al. Spatial patterns of incident malaria cases and their household contacts in a single clinic catchment area of Chongwe District, Zambia. *Malar J* 2015; 14:305.**
151. *Pongvongsa T, Nonaka D, Iwagami M, Nakatsu M, Phongmany P, et al. Household clustering of asymptomatic malaria infections in Xepon district, Savannakhet province, Lao PDR. Malar J 2016; 15:508.*
152. *Por I, Sovannaroth S, Moran A, Dysoley L, Nguon S, et al. Cost-effectiveness of malaria elimination in Sampov Luon Operational District, Cambodia. Malariaworld J 2020; 11: 2.*
153. *Pradhan MM, Anvikar AR, Daumerie PG, Pradhan S, Dutta A, et al. Comprehensive case management of malaria: operational research informing policy. J Vector Dis 2019; 56(1): 56-59.*
154. *Pringle JC, Tessema S, Wesolowski A, Chen A, Murphy M, et al. Genetic evidence of focal Plasmodium falciparum transmission in a pre-elimination setting in Southern Province, Zambia. J Infect Dis 2019; 219(8):1254-1263.*
155. *Rajakaruna RS, Alifrangis M, Amerasinghe PH, Konradsen F. Pre-elimination stage of malaria in Sri Lanka: assessing the level of hidden parasites in the population. Malar J 2010; 9:25.*
156. *Rajvanshi H, Bharti PK, Nisar S, Jain Y, Jayswar H, et al. Study design and operational framework for a community-based Malaria Elimination Demonstration Project (MEDP) in 1233 villages of district Mandla, Madhya Pradesh. Malar J 2020; 19:410.*
157. *Ray AP. The discipline and dynamics of active case detection procedure under surveillance operations in a malaria eradication programme. WHO/Mal/453. Geneva: World Health Organization; 1964.*
158. **Reiker T, Chitnis N, Smith T. Modelling reactive case detection strategies for interrupting transmission of *Plasmodium falciparum* malaria. *Malar J* 2019; 18:259.**
159. *Roberts KW, Gueye CS, Baltzell K, Ntuku H, McCreesh P, et al. Community acceptance of reactive focal mass drug administration and reactive focal vector control using indoor residual spraying, a mixed-methods study in Zambezi region, Namibia. Malar J 2021; 20:162.*
160. **Rogawski ET, Congpuong K, Sudathip P, Satimai W, Sug-aram R, et al. Active case detection with pooled real-time PCR to eliminate malaria in Trat Province, Thailand. *Am J Trop Med Hyg* 2012; 86(5): 789-791.**
161. *Rosas-Aguirre A, Speybroeck N, Llanos-Cuentas A, Rosanas-Urgell A, Carrasco-Escobar G, et al. Hotspots of malaria transmission in the Peruvian Amazon: rapid assessment through a parasitological and serological survey. PLoS ONE 2015; 10(9): e0137458.*
162. *Rosas-Aguirre A, Llanos-Cuentas A, Speybroeck N, Cook J, Contreras-Mancilla J, et al. Assessing malaria transmission in a low endemicity area of north-western Peru. Malar J 2013; 12:339.*
163. **Rosas-Aguirre A, Erhart A, Llanos-Cuentas A, Branch O, Berkvens D, et al. Modelling the potential of focal screening and treatment as elimination strategy for *Plasmodium falciparum* malaria in the Peruvian Amazon Region. *Parasit Vectors* 2015; 8:261.**
164. **Rossi G, Van den Bergh R, Nguon C, Debackere M, Vernaeve L, et al. Adapting reactive case detection strategies for falciparum malaria in a low-transmission area in Cambodia. *Clin Infect Dis* 2018; 66(2): 296-298.**
165. **Rossi G, Vernaeve L, Van den Bergh R, Nguon C, Debackere M, et al. Closing in on the reservoir: proactive case detection in high-risk groups as a strategy to detect Plasmodium falciparum asymptomatic carriers in Cambodia. *Clin Infect Dis* 2018; 66(10): 1610-1617.**
166. *Ruktanonchai NW, DeLeenheer P, Tatem AJ, Alegana VA, Caughlin TT, et al. Identifying malaria transmission foci for elimination using human mobility data. PLoS Comput Biol 2016; 12(4): e1004846.*
167. **Rulisa S, Kateera F, Bizimana JP, Agaba S, Dukuzumuremyi J, et al. Malaria prevalence, spatial clustering and risk factors in a low endemic area of Eastern Rwanda: a cross sectional study. *PLoS ONE* 2013; 8(7): e69443.**
168. **Samuels AM, Odero NA, Odongo W, Otieno K, Were V, et al. Impact of community-based mass testing and treatment on malaria infection prevalence in a high-transmission area of western Kenya: a cluster randomized controlled trial. *Clin Infect Dis* 2021; 72(11): 1927-1935.**
169. *Sanders K, Smith Gueye C, Phillips AA, Gosling R. Active case detection for malaria elimination: a confusion of acronyms and definitions. Malar Chemother Control Elimin 2012; 1(1): 1-5.*
170. **Scott CA, Yeshiwondim AK, Serda B, Guinovart C, Tesfay BH, et al. Mass testing and treatment for malaria in low transmission areas in Amhara Region, Ethiopia. *Malar J* 2016; 15:305.**
171. **Searle KM, Hamapumbu H, Lubinda J, Shields TM, Pinchoff J, et al. Evaluation of the operational challenges in implementing reactive screen-and-treat and implications of reactive case detection strategies for malaria elimination in a region of low transmission in southern Zambia. *Malar J* 2016; 15:412.**
172. **Searle KM, Shields T, Hamapumbu H, Kobayashi T, Mharakurwa S, et al. Efficiency of household reactive case detection for malaria in rural southern Zambia: simulations based on cross-sectional surveys from two epidemiological settings. *PLoS ONE* 2013; 8(8): e70972.**
173. **Searle KM, Katowa B, Musonda M, Pringle JC, Hamapumbu H, et al. Sustained malaria transmission despite reactive screen-and-treat in a low-transmission area of southern Zambia. *Am J Trop Med Hyg* 2021; 104(2): 671.**
174. *Searle KM, Katowa B, Kobayashi T, Siame MNS, Mharakurwa S, et al. Distinct parasite populations infect individuals identified through passive and active case detection in a region of declining malaria transmission in southern Zambia. Malar J 2017; 16:154.*
175. *Shuford K, Were F, Awino N, Samuels A, Ouma P, et al. Community perceptions of mass screening and treatment for malaria in Siaya County, western Kenya. Malar J 2016; 15:71.*
176. **Silal SP, Little F, Barnes KI, White LJ. Hitting a moving target: a model for malaria elimination in the presence of population movement. *PLoS ONE* 2015; 10(12): e0144990.**
177. **Silal SP, Little F, Barnes KI, White LJ. Predicting the impact of border control on malaria transmission: a simulated focal screen and treat campaign. *Malar J* 2015; 14:268.**
178. **Silal SP, Little F, Barnes KI, White LJ. Towards malaria elimination in Mpumalanga, South Africa: a population-level mathematical modelling approach. *Malar J* 2014; 13:297.**
179. *Silumbe K, Yukich JO, Hamainza B, Bennett A, Earle D, et al. Costs and cost-effectiveness of a large-scale mass testing and treatment intervention for malaria in Southern Province, Zambia. Malar J 2015; 14:211.*
180. *Silumbe K, Chiyende E, Finn TP, Desmond M, Puta C, et al. A qualitative study of perceptions of a mass test and treat campaign in Southern Zambia and potential barriers to effectiveness. Malar J 2015; 14:171.*
181. *Singh N, Bharti PK, Kumre NS. Active v. passive surveillance for malaria in remote tribal belt of Central India: implications for malaria elimination. Pathog Glob Health 2016; 110(4-5): 178-184.*
182. **Slater HC, Ross A, Ouedraogo AL, White LJ, Nguon C, et al. Assessing the impact of next-generation rapid diagnostic tests on *Plasmodium falciparum* malaria elimination strategies. *Nature* 2015; 528: S94-S101.**
183. **Smith JL, Auala J, Tambo M, Haindongo E, Katokele S, et al. Spatial clustering of patent and sub-patent malaria infections in northern Namibia: implications for surveillance and response strategies for elimination. *PLoS ONE* 2017; 12(8): e0180845.**
184. *Smith Gueye C, Sanders KC, Galappaththy GNL, Rundi C, Tobgay T, et al. Active case detection for malaria elimimation: a survey among Asia Pacific countries. Malar J 2013; 12:358.*
185. **Stratil AS, Vernaeve L, Lopes S, Bourny Y, Mannion K, et al. Eliminating *Plasmodium falciparum* malaria: results from tailoring active case detection approaches to remote populations in forested border areas in north-eastern Cambodia. *Malar J* 2021; 20:108.**
186. **Stresman GH, Kamanga A, Moono P, Hamapumbu H, Mharakurwa S, et al. A method of active case detection to target reservoirs of asymptomatic malaria and gametocyte carriers in a rural area in Southern Province, Zambia. *Malar J* 2010; 9:265.**
187. **Stresman GH, Baidjoe AY, Stevenson J, Grignard L, Odongo W, et al. Focal screening to identify the subpatent parasite reservoir in an area of low and heterogeneous transmission in the Kenya highlands. *J Infect Dis* 2015; 212(11): 1768-1777.**
188. *Stresman G, Bousema T, Cook J. Malaria hotspots: is there epidemiological evidence for fine-scale spatial targeting of interventions? Trends Parasitol 2019; 35(10):822-834.*
189. *Stresman G, Whittaker C, Slater HC, Bousema T, Cook J. Quantifying Plasmodium falciparum infections clustering within households to inform household-based intervention strategies for malaria control programs: an observational study and meta-analysis from 41 malaria-endemic countries. PLoS Med 2020; 17(10): e1003370.*
190. **Stuck L, Fakih BS, Abdul-wahid H, Hofmann NE, Holzschuh A, et al. Malaria infection prevalence and sensitivity of reactive case detection in Zanzibar. *Int J Infect Dis* 2020; 97: 337-346.**
191. **Stuckey EM, Miller JM, Littrell M, Chitnis N, Steketee R. Operational strategies of anti-malarial drug campaigns for malaria elimination in Zambia’s Southern Province; a simulation study. *Malar J* 2016; 15:148.**
192. **Sturrock HJW, Novotny JM, Kunene S, Dlamini S, Zulu Z, et al. Reactive case detection for malaria elimination: real-life experience from an ongoing program in Swaziland. *PLoS ONE* 2013; 8(5): e63830.**
193. *Sturrock HJW, Hsiang MS, Cohen JM, Smith DL, Greenhouse B, et al. Targeting asymptomatic malaria infections: active surveillance in control and elimination. PLoS Med 2013; 10(6): e1001467.*
194. **Sutanto I, Kosasih A, Elyazar IRF, Simanjuntak DR, Larasati TA, et al. Negligible impact of mass screening and treatment on meso-endemic malaria transmission at West Timor in Eastern Indonesia: a cluster-randomised trial. *Clin Infect Dis* 2018; DOI: 10.1093/cid/ciy231.**
195. **Sutcliffe CG, Kobayashi T, Hamapumbu H, Shields T, Mharakurwa S, et al. Reduced risk of malaria parasitemia following household screening and treatment: a cross-sectional and longitudinal cohort study. *PLoS ONE* 2012; 7(2): e31396.**
196. *Sutcliffe CG, Kobayashi T, Hamapumbu H, Shields T, Kamanga A, et al. Changing individual-level risk factors for malaria with declining transmission in southern Zambia: a cross-sectional study. Malar J 2011; 10:324.*
197. *Tadesse FG, Pett H, Baidjoe A, Lanke K, Grignard L, et al. Submicroscopic carriage of Plasmodium falciparum and Plasmodium vivax in a low endemic area in Ethiopia where no parasitaemia was detected by microscopy or rapid diagnostic test. Malar J 2015; 14:303.*
198. *Taffon P, Rossi G, Kindermans J-M, Van den Bergh R, Nguon C, et al. ‘I could not join because I had to work for pay.’: a qualitative evaluation of falciparum malaria pro-active case detection in three rural Cambodian villages. PLoS ONE 2018; 13(4):e0195809.*
199. *Tambo M, Auala JR, Sturrock HJ, Kleinschmidt I, Bock R, et al. Evaluation of loop-mediated isothermal amplification as a surveillance tool for malaria in reactive case detection moving towards elimination. Malar J 2018; 17:255.*
200. *Tatarsky A, Aboobakar S, Cohen JM, Gopee N, Bheecarry A, et al. Preventing the reintroduction of malaria in Mauritius: a programmatic and financial assessment. PLoS ONE 2011; 6(9): e23832.*
201. **Tejedor-Garavito N, Dlamini N, Pindolia D, Soble A, Ruktanonchai NW, et al. Travel patterns and demographic characteristics of malaria cases in Swaziland, 2010-2014. *Malar J* 2017; 16:359.**
202. **Tessema SK, Belachew M, Koepfli C, Lanke K, Huwe T, et al. Spatial and genetic clustering of *Plasmodium falciparum* and *Plasmodium vivax* infections in a low-transmission area of Ethiopia. *Sci Rep* 2020; 10:19975.**
203. *Thanh PV, Hong NV, Van NV, Malderen CV, Obsomer V, et al. Epidemiology of forest malaria in Central Vietnam: the hidden parasite reservoir. Malar J 2015: 14:86.*
204. *Tietje K, Hawkins K, Clerk C, Ebels K, McGray S, et al. The essential role of infection-detection technologies for malaria elimination and eradication. Trends Parasitol 2014; 30(5): 259-266.*
205. *Tiono AB, Kangoye DT, Rehman AM, Kargougou DG, Kabore Y, et al. Malaria incidence in children in South-West Burkina Faso: comparison of active and passive case detection methods. PLoS ONE 2014; 9(1): e86936.*
206. **Tiono AB, Ouedraogo A, Ogutu B, Diarra A, Coulibaly S, et al. A controlled, parallel, cluster-randomized trial of community-wide screening and treatment of asymptomatic carriers of *Plasmodium falciparum* in Burkina Faso. *Malar J* 2013; 12:9.**
207. *Tseroni M, Baka A, Kapizioni C, Snounou G, Tsiodras S, et al. Prevention of malaria resurgence in Greece through the association of mass drug administration (MDA) to immigrants from malaria-endemic regions and standard control measures. PLoS Negl Trop Dis 2015; 9(11): e0004215.*
208. *Tseroni M, Georgitsou M, Baka A, Pinaka O, Pervanidou D, et al. The importance of an active case detection (ACD) programme for malaria among migrants from malaria endemic countries: the Greek experience in a receptive and vulnerable area. Int J Environ Res Public Health 2020; 17(11): 4080.*
209. *Tsoka-Gwegweni JM, Okafor U. Asymptomatic malaria in refugees living in a non-endemic South African city. PLoS ONE 2014; 9(9): e107693.*
210. **Tun STT, von Seidlein L, Pongvongsa T, Mayxay M, Saralamba S, et al. Towards malaria elimination in Savannakhet, Lao PDR: mathematical modelling driven strategy design. *Malar J* 2017; 16(1):483.**
211. *Turki H, Raeisi A, Malekzadeh K, Ghanbarnejad A, Zoghi S, et al. Efficiency of nested-PCR in detecting asymptomatic cases toward malaria elimination program in an endemic area of Iran. Iran J Parasitol 2015; 10(1): 39-45.*
212. *Vallejo AF, Chaparro PE, Benavides Y, Alvarez A, Quintero JP, et al. High prevalence of sub-microscopic infections in Colombia. Malar J 2015; 14:201.*
213. *Vallejo AF, Martinez NL, Gonzalez IJ, Arevalo-Herrera M, Herrera S. Evaluation of the loop mediated isothermal DNA amplification (LAMP) kit for malaria diagnosis in P. vivax endemic settings of Colombia. PLoS Negl Trop Dis 2015; 9(1): e3453.*
214. *van der Horst T, Al-Mafazy AW, Fakih BS, Stuck L, Ali A, Yukich J, Hetzel MW. Operational coverage and timeliness of reactive case detection for malaria elimination in Zanzibar, Tanzania. Am J Trop Med Hyg 2020; 102(2): 298.*
215. **van Eijk AM, Ramanathapuram L, Sutton PL, Kanagaraj D, Priya GSL, et al. What is the value of reactive case detection in malaria control? A case-study in India and a systematic review. *Malar J* 2016; 15:67.**
216. *Vasquez-Jimenez JM, Arevalo-Herrera M, Henao-Giraldo J, Molina-Gomez K, Arce-Plata M, et al. Consistent prevalence of asymptomatic infections in malaria endemic populations in Colombia over time. Malar J 2016; 15:70.*
217. **Vilakati S, Mngadi N, Benjamin-Chung J, Dlamini N, Dufour MS, et al. Effectiveness and safety of reactive focal mass drug administration (rfMDA) using dihydroartemisinin-piperaquine to reduce malaria transmission in a very low-endemic setting of Eswatini: a pragmatic cluster randomised controlled trial. *BMJ Glob Health* 2021; 6: e005021.**
218. *Vitor-Silva S, Siqueira AM, Sampaio VS, Guinovart C, Reyes-Lecca RC, et al. Declining malaria transmission in rural Amazon: changing epidemiology and challenges to achieve elimination. Malar J 2016; 15:266.*
219. *von Seidlein, L. The failure of screening and treating as a malaria elimination strategy. PLoS Med 2014; 11(1): e1001595.*
220. **Wang D, Cotter C, Sun X, Bennett A, Gosling RD, Xiao N. Adapting the local response for malaria elimination through evaluation of the 1-3-7 system performance in the China-Myanmar border region. *Malar J* 2017; 16:54.**
221. *Wang CM, Hu SC, Hung WS, Chang HL, Wu HS, et al. The absence of endemic malaria transmission in Taiwan from 2002 to 2010: the implications of sustained malaria elimimation in Taiwan. Travel Med Infect Dis 2012; 10(5-6): 240-246.*
222. *Wangdi K, Banwell C, Gatton ML, Kelly GC, Namgay R, Clements ACA. Development and evaluation of a spatial decision support system for malaria elimination in Bhutan. Malar J 2016; 15:180.*
223. *Wen S, Harvard KE, Smith Gueye C, Canavati SE, Chancellor A, et al. Targeting populations at higher risk for malaria: a survey of national malaria elimination programmes in the Asia Pacific. Malar J 2016; 15:271.*
224. *West N, Gyeltshen S, Dukpa S, Khoshnood K, Tashi S, et al. An evaluation of the national malaria surveillance system of Bhutan, 2006-2012 as it approaches the goal of malaria elimination. Front Public Health 2016; 4:167.*
225. *Wickremasinghe R, Fernando SD, Thillekaratne J, Wijeyaratne PM, Wickremasinghe AR. Importance of active case detection in a malaria elimination programme. Malar J 2014; 13:186.*
226. *Wu L, van den Hoogen LL, Slater H, Walker PGT, Ghani AC, et al. Comparison of diagnostics for the detection of asymptomatic Plasmodium falciparum infections to inform control and elimination strategies. Nature 2015; 528:S86-S93.*
227. **Yukich J, Bennett A, Yukich R, Stuck L, Hamainza B, et al. Estimation of malaria parasite reservoir coverage using reactive case detection and active community fever screening from census data with rapid diagnostic tests in southern Zambia: a re-sampling approach. *Malar J* 2017; 16:317.**
228. *Zaw MT, Thant M, Hlaing TM, Aung NZ, Thu M, et al. Asymptomatic and sub-microscopic malaria infection in Kayah State, eastern Myanmar. Malar J 2017; 16:138.*
229. **Zelman BW, Baral R, Zarlinda I, Coutrier FN, Sanders KC, et al. Costs and cost-effectiveness of malaria reactive case detection using loop-mediated isothermal amplification compared to microscopy in the low transmission setting of Aceh Province, Indonesia. *Malar J* 2018; 17:220.**
230. **Zemene E, Koepfli C, Tiruneh A, Yeshiwondim AK, Seyoum D, et al. Detection of foci of residual malaria transmission through reactive case detection in Ethiopia. *Malar J* 2018; 17:390.**
231. *Zeng XC, Sun XD, Li JX, Chen MN, Deng DW, et al. Assessment of malaria control consultation and service posts in Yunnan, P.R. China. Infect Dis Poverty 2016; 5:102.*
232. **Zhang X, Yao L, Sun J, Pan J, Chen H, Zhang L, Ruan W. Malaria in Southeastern China from 2012 to 2016: analysis of imported cases. *Am J Trop Med Hyg* 2018; 98(4): 1107-1112.**
233. *Zhou SS, Zhang SS, Zhang L, Rietveld AEC, Ramsay AR, et al. China’s 1-3-7 surveillance and response strategy for malaria elimination: is case reporting, investigation and foci response happening according to plan? Infect Dis Poverty 2015; 4:55.*
234. *Zhu M, Ruan W, Fei SJ, Song JQ, Zhang Y, et al. Approaches to the evaluation of malaria elimination at county level: case study in the Yangtze River Delta. Adv Parasitol 2014; 86:135-182.*
235. *Zoghi S, Mehrizi AA, Raeisi A, Haghdoost AA, Turki H, et al. Survey for asymptomatic malaria cases in low transmission settings of Iran under elimination programme. Malar J 2012; 11:126.*

## Appendix D

##

## Appendix E. Summary of observational proactive MTaT studies (n=15)

The 15 MTaT observational studies were pilots or reports of program experience with MTaT and aimed to assess impact on transmission, yield and operational feasibility, including with different diagnostic approaches and proactive TTaT, or targeted testing and treatment of high risk populations

Six studies aimed to measure impact of MTaT on transmission reduction through pre-post or quasi-experimental designs:

- In a moderate transmission setting in Zambia, a quasi-experimental study was used to compare one versus five rounds of MTaT over one year, as well as one versus 10 rounds over two years. Parasite prevalence by RDT was significantly lower in areas that received multiple MTaT rounds, and effects were greater in the area with lower baseline prevalence (a six- versus two-fold reduction was observed when baseline prevalence was 4% versus 24%). However, there was only one cluster per arm, prevalence was not measured concurrently in the comparison arms, and effects may have been overestimated because households were repeatedly surveyed, and their malaria prevention behaviors may have been influenced by study participation.^[[2]](#footnote-2)^
- On Principe island, a very low transmission setting, three rounds of annual, island-wide MTaT targeting ~6,000 individuals were carried out by the malaria program as part of a package of pre-elimination interventions that included vector control, intermittent preventative therapy for pregnant women, and case management. The decline in incidence from 16 per 100 population after other interventions were already in place to 0.7 per 100 population with addition of MTaT suggested that MTaT was a critical component, but ecological effects could not be ruled out.^[[3]](#footnote-3)^
- In a high transmission setting in Ghana, four rounds of MTaT conducted over one year led to a 24% decline in parasite prevalence, and 9% reduction in symptomatic parasitemia. However, the pre-post analysis did not account for potential ecological effects.^[[4]](#footnote-4)^
- In a quasi-experimental study from a low transmission setting in Senegal, MTaT followed by weekly testing and treatment for fever cases by community health workers led to a 38% decrease in malaria incidence relative to the comparison group, after adjusting for environmental factors and vector control. However, the effect was mainly attributed to the program of weekly testing and treatment for fever cases.^[[5]](#footnote-5)^
- In a very low transmission setting in India, malaria cases decreased by 91% after three rounds of MTaT, but intensive case management and vector control were implemented at the same time and the study could not control for ecological factors.^[[6]](#footnote-6)^
- In a low and heterogeneous transmission setting in the Kenya highlands, the use of RDT to inform household-level treatment was evaluated to gauge the efficiency of a future MTaT+fMDA approach. RDT-positive households included 77% of PCR-positive individuals but the subsequent MTaT+fMDA trial did not show notable impact.^[[7]](#footnote-7)^

Seven studies from low and very low transmission settings evaluated the yield or operational feasibility of different diagnostic approaches. In studies from Cambodia,^[[8]](#footnote-8),^^[[9]](#footnote-9),^^[[10]](#footnote-10)^ Kenya,^7^ Peru,^[[11]](#footnote-11)^ Vanuatu,^[[12]](#footnote-12)^ and Zanzibar,^[[13]](#footnote-13)^ the use of molecular methods yielded positivity rates from under 1% to 54.3% and increased detection of infections by two- to 17-fold compared to RDT. The highest fold increase was for detection of *P. vivax* infection by RDT versus PCR.^10^ Three of these studies used molecular testing results in real-time to guide treatment.^8,9,13^ In Cambodia, MTaT using PCR was evaluated, but the turnaround time between sample collection and treatment was high at 8 days. It was noted that a mobile lab would help overcome some of the delays and logistical challenges inherent in PCR-based diagnosis.^8^ A more recent study from Cambodia also noted long turnaround times for PCR and called for more sensitive point-of-care diagnostics.^9^ However, in Zanzibar, LAMP (a molecular test that can be conducted with a 3-hour turnaround time) was deemed neither field-friendly nor operationally feasible as a programmatic tool due to cost and complexity of the methods.^13^

Overall operational feasibility of MTaT was assessed in two studies:

- A program experience report from a low transmission region in Ethiopia found that one round of MTaT in approximately 30,000 individuals yielded a 1.4% RDT positivity rate and no clear risk factors for infection were identified. The staff and training needs for this pilot were believed to exceed existing programmatic capacity.^[[14]](#footnote-14)^
- In a low transmission setting in Peru, multiple rounds of microscopy-based MTaT (one round every 10 days, four rounds total) among the same target population detected five times more cases versus a single round, but this approach was less effective in detecting cases compared to a single round of PCR-based MTaT. Study authors emphasized the need for a more detailed feasibility and cost-effectiveness assessment based on the different operational criteria of the various approaches.^[[15]](#footnote-15)^

Three studies in low and very low transmission settings in the Greater Mekong Subregion evaluated MTaT using additional delivery methods to address the challenge of reaching mobile populations and migrant workers:

- In Cambodia, proactive case detection was conducted in villages in conjunction with pre-intervention social mobilization and promotion, and attendance by at-risk and mobile populations was consistently high across three rounds of proactive MTaT using PCR.^9^
- Another study from Cambodia targeted high risk forest and plantation workers through village-level proactive case detection with molecular testing, whereby health promotion officers conducted outreach and testing. This approach identified twice as many *P. falciparum* infections compared to standard passive case detection and reactive case detection.^[[16]](#footnote-16)^
- In Myanmar, testing through mobile clinics and village-level malaria workers (using standard diagnostics) was compared to fixed screening points in border areas with high traffic of mobile and migrant populations. Voluntary testing at fixed screening points tested fewer people, but overall yielded relatively high positivity rates because the volunteers typically had fever or other symptoms that led them to seek care. A noted benefit of fixed screening points was their ability to reach populations that are otherwise difficult to reach.^[[17]](#footnote-17)^

**Appendix F. Detail of RACD studies (n=47)**

Appendix G: Reactive case detection (RACD) Positivity (%) by Rapid diagnostic test (RDT) or microscopy

Appendix H: Relative difference in reactive case detection (RACD) positivity by LAMP/PCR versus RDT/microscopy. Summary estimates are reported by transmission strata and generated using a random effects model.

1. Global Malaria Programme. A framework for malaria elimination. 2017. http://www.who.int/malaria/publications/atoz/WHO-malaria-elimination-framework-2017-presentation-en.pdf (accessed Nov 27, 2021). [↑](#footnote-ref-1)
2. Sutcliffe CG, Kobayashi T, Hamapumbu H, Shields T, Mharakurwa S, et al. Reduced risk of malaria parasitemia following household screening and treatment: a cross-sectional and longitudinal cohort study. *PLoS ONE* 2012; 7(2): e31396. [↑](#footnote-ref-2)
3. Lee PW, Liu CT, Rampao HS, do Rosario VE, Shaio MF. Pre-elimination of malaria on the island of Principe. *Malar J* 2010; 9:26. [↑](#footnote-ref-3)
4. Ndong IC, Okyere D, Enos JY, Mensah BA, Nyarko A, et al. Prevalence of asymptomatic malaria parasitaemia following mass testing and treatment in Pakro sub-district of Ghana. *BMC Pub Health* 2019; 19:1622. [↑](#footnote-ref-4)
5. Conner RO, Dieye Y, Hainsworth M, Tall A, Cisse B, et al. Mass testing and treatment for malaria followed by weekly fever screening, testing and treatment in Northern Senegal: feasibility, cost and impact. *Malar J* 2020; 19:252. [↑](#footnote-ref-5)
6. Bharti PK, Rajvanshi H, Nisar S, Jayswar H, Saha KB, et al. Demonstration of indigenous malaria elimination through Track-Test-Treat-Track (T4) strategy in a Malaria Elimination Demonstration Project in Mandla, Madhya Pradesh. *Malar J* 2020; 19:339. [↑](#footnote-ref-6)
7. Stresman GH, Baidjoe AY, Stevenson J, Grignard L, Odongo W, et al. Focal screening to identify the subpatent parasite reservoir in an area of low and heterogeneous transmission in the Kenya highlands. *J Infect Dis* 2015; 212(11): 1768-1777. [↑](#footnote-ref-7)
8. Hoyer S, Nguon S, Kim S, Habib N, Khim N, et al. Focused screening and treatment (FSAT): a PCR-based strategy to detect malaria parasite carriers and contain drug resistant *P. falciparum*, Pailin, Cambodia. *PLoS ONE* 2012; 7(10): e45797. [↑](#footnote-ref-8)
9. Rossi G, Vernaeve L, Van den Bergh R, Nguon C, Debackere M, et al. Closing in on the reservoir: proactive case detection in high-risk groups as a strategy to detect Plasmodium falciparum asymptomatic carriers in Cambodia. *Clin Infect Dis* 2018; 66(10): 1610-1617. [↑](#footnote-ref-9)
10. Kunkel A, Nguon C, Iv S, Chhim S, Peov D, et al. Choosing interventions to eliminate forest malaria: preliminary results of two operational research studies inside Cambodian forests. *Malar J* 2021; 20:51. [↑](#footnote-ref-10)
11. Moreno-Gutierrez D, Llanos-Cuentas A, Barboza JL, Contreras-Mancilla J, Gamboa D, et al. Effectiveness of a malaria surveillance strategy based on active case detection during high transmission season in the Peruvian Amazon. *Int J Environ Res Public Health* 2018; 15(12):2670. [↑](#footnote-ref-11)
12. Donald W, Pasay C, Guintran JO, Iata H, Anderson K, et al. The utility of malaria rapid diagnostic tests as a tool in enhanced surveillance for malaria elimination in Vanuatu. *PLoS ONE* 2016; 11(11): e0167136. [↑](#footnote-ref-12)
13. Cook J, Aydin-Schmidt B, Gonzalez IJ, Bell D, Edlund E, et al. Loop-mediated isothermal amplification (LAMP) for point-of-care detection of asymptomatic low-density malaria parasite carriers in Zanzibar. *Malar J* 2015; 14:43. [↑](#footnote-ref-13)
14. Scott CA, Yeshiwondim AK, Serda B, Guinovart C, Tesfay BH, et al. Mass testing and treatment for malaria in low transmission areas in Amhara Region, Ethiopia. *Malar J* 2016; 15:305. [↑](#footnote-ref-14)
15. Moreno-Gutierrez D, Llanos-Cuentas A, Barboza JL, Contreras-Mancilla J, Gamboa D, et al. Effectiveness of a malaria surveillance strategy based on active case detection during high transmission season in the Peruvian Amazon. *Int J Environ Res Public Health* 2018; 15(12):2670. [↑](#footnote-ref-15)
16. Stratil AS, Vernaeve L, Lopes S, Bourny Y, Mannion K, et al. Eliminating *Plasmodium falciparum* malaria: results from tailoring active case detection approaches to remote populations in forested border areas in north-eastern Cambodia. *Malar J* 2021; 20:108. [↑](#footnote-ref-16)
17. Kheang ST, Lin MA, Lwin S, Naing YH, Yarzar P, Kak N, Price T. Malaria case detection among mobile populations and migrant workers in Myanmar: comparisons of 3 service delivery approaches. *Glob Health Sci Pract* 2018; 6(2):384-389. [↑](#footnote-ref-17)
